# Meta-analysis on reporting practices as a source of heterogeneity in in vitro cancer research

**DOI:** 10.1136/bmjos-2021-100272

**Published:** 2022-06-01

**Authors:** Timo Sander, Joly Ghanawi, Emma Wilson, Sajjad Muhammad, Malcolm Macleod, Ulf Dietrich Kahlert

**Affiliations:** 1 Department of Neurosurgery, Medical Faculty, Heinrich Heine University, Düsseldorf, Germany; 2 Independent researcher, Edinburgh, UK; 3 Centre for Clinical Brain Sciences, The University of Edinburgh Medical School, Edinburgh, UK; 4 Department of Molecular and Experimental Surgery, Clinic for General, Visceral, Vascular and Transplant Surgery, Otto von Guericke Universität Magdeburg, Magdeburg, Germany

**Keywords:** Biomedical Research, Translational Medical Research, Cell Proliferation

## Abstract

**Objectives:**

Heterogeneity of results of exact same research experiments oppose a significant socioeconomic burden. Insufficient methodological reporting is likely to be one of the contributors to results heterogeneity; however, little knowledge on reporting habits of in vitro cancer research and their effects on results reproducibility is available. Exemplified by a commonly performed in vitro assay, we aim to fill this knowledge gap and to derive recommendations necessary for reproducible, robust and translational preclinical science.

**Methods:**

Here, we use systematic review to describe reporting practices in in vitro glioblastoma research using the Uppsala-87 Malignant Glioma (U-87 MG) cell line and perform multilevel random-effects meta-analysis followed by meta-regression to explore sources of heterogeneity within that literature, and any associations between reporting characteristics and reported findings. Literature that includes experiments measuring the effect of temozolomide on the viability of U-87 MG cells is searched on three databases (Embase, PubMed and Web of Science).

**Results:**

In 137 identified articles, the methodological reporting is incomplete, for example, medium glucose level and cell density are reported in only 21.2% and 16.8% of the articles. After adjustments for different drug concentrations and treatment durations, the results heterogeneity across the studies (I^2^=68.5%) is concerningly large. Differences in culture medium glucose level are a driver of this heterogeneity. However, infrequent reporting of most experimental parameters limits the analysis of reproducibility moderating parameters.

**Conclusions:**

Our results further support the ongoing efforts of establishing consensus reporting practices to elevate durability of results. By doing so, this work can raise awareness of how stricter reporting may help to improve the frequency of successful translation of preclinical results into human application. The authors received no specific funding for this work. A preregistered protocol is available at the Open Science Framework (https://osf.io/9k3dq).

**Strengths and limitations of the study**

- This is a novel systematic review and meta-analysis focusing on the reproducibility of a commonly performed in vitro drug test assay in cancer research, including a large sample size of evidence with 137 articles.
- We used a three-level meta-analytical random-effects model accounting for dependence of data from the same article.
- Full availability of the statistical code used and the underlying data is offered for transparent methods.
- The statistical power in meta-regressions investigating heterogeneity moderators is limited due to infrequent reporting of experimental parameters.
- We experienced a low rate of response (8.0%) when asking the authors of the included articles for additional information regarding unreported parameters.

## Introduction

Progress in scientific research is a dynamic process which thrives in the interaction of diverse research groups addressing shared problems. The scientific model has new findings either confirmed or refuted by other scientists, so that science becomes self-correcting.^1^ However, one key foundation for self-correction is that key experimental methods needed for interpretation and repetition of published research are described in sufficient detail. Recent efforts to replicate key findings in cancer biology and further research areas have raised scientific, ethical and economic concerns.^2–9^

Glioblastoma is a malignant brain tumour with a median time of survival of around 15 months.^10^ First-line treatment consists of a multimodal approach of surgical resection followed by radiation therapy and chemotherapy with the alkylating agent temozolomide (TMZ).^11^ A previous systematic review of the in vivo literature describing the efficacy of TMZ showed limited reporting of key study design features and low prevalence of reporting of measures to reduce risks of bias.^12^ In vitro glioblastoma research commonly uses the commercially available cell line Uppsala-87 Malignant Glioma (U-87 MG),^13 14^ originally derived in 1966 from a female patient at Uppsala University.^15^ However, the currently available U-87 MG line distributed by the American Type Culture Collection (ATCC, Manassas, Virginia, USA)^16^ has been found to be different from the original version.^17^ It is unclear to what extent these U-87 MG cells are truly representative of the original tumour tissue and whether they allow for reproducible experiments when serving as glioblastoma models.

The reproducibility of in vitro glioma research is likely to depend on the completeness of reporting of key study design features, including reporting of risks of bias. Here, we use a systematic review to portray the in vitro literature describing the effectiveness of TMZ in reducing the growth of U-87 MG cells, with a focus on the use of clinically relevant drug concentrations and treatment durations, on methodological reporting and how this might influence the reproducibility of results.

## Methods

A preregistered study protocol is available at the Open Science Framework^18^ and was uploaded before full text based screening and data extraction began. Deviations from the protocol are described at the appropriate sections.

## Systematic review

### Systematic literature search and screening

The systematic search was conducted on the databases PubMed, Embase and Web of Science on 26 August 2020 using the search strategy described in the online supplemental additional data A1.^19^ Two reviewers independently screened article titles and abstracts for potential inclusion, with discrepancies resolved by a third reviewer. This was followed by full text screening. We included studies describing controlled in vitro cell culture experiments that compared the effect of a single TMZ treatment on the viability of U-87 MG cells with that in untreated controls. We also required that cell viability was measured by colorimetric assay or by cell counting, and that the authors used Dulbecco’s Modified Eagle Medium (DMEM) as the ATCC recommends using a modified Eagle medium for U-87 MG cell cultures^16^ and as a recent review identified DMEM as the most frequently used medium for this type of experiment.^14^ This culture medium restriction was introduced to guarantee sufficient comparability of experiments across studies and to prevent interference of different culture media in the analysis of the impact of culture medium ingredients (eg, glucose concentration and antibiotics) on the overall effect and its reproducibility. We only included original peer-reviewed research articles in the English language, with no restrictions made on the publication year. Our protocol had included consideration of cell growth rates in xenotransplantation models, but we later decided to focus exclusively on in vitro research. Detailed inclusion and exclusion criteria are given in online supplemental additional data A2.^19^

10.1136/bmjos-2021-100272.supp1 Supplementary data

### Data extraction

We recorded effect sizes for change in cell viability in response to TMZ compared with untreated control, TMZ concentration and duration of exposure. As shown in online supplemental additional data A3 and A4,^19^ we recorded 18 experimental parameters, 8 risks of bias items and the journal impact factor (JIF) for that journal in the year of publication.^20^ Consideration of JIF had not been included in our study protocol and should be considered an exploratory analysis.

Two reviewers independently recorded study design features, risk of bias items and effect sizes, with reconciliation of discrepancies by a third reviewer where necessary; data for effect sizes were reconciled if they differed by more than 15% of the largest effect size; otherwise, the mean of the extracted values from the two reviewers was used. Where information was missing, we contacted authors for clarification; to reduce the burden on them to reply, we limited this request to a maximum of 11 items as described in the online supplemental additional data A5.^19^

### Reporting quality of experimental parameters and articles

Where an article reported multiple relevant experiments, a parameter was considered as reported for an article if it was reported in every belonging experiment. When calculating the number of items reported for each article, we did not consider the volume of added TMZ and of control fluid as we considered this was included if the drug and control concentration was given. We analysed change in reporting quality over time, and any relationship between reporting quality and JIF, using linear regression.

## Meta-analysis

### Exclusions from the meta-analysis

We excluded experiments that did not report data essential for our analysis such as cell viability data for the untreated control group, TMZ concentration, duration of treatment or the number of experimental units; and we excluded baseline data (where treatment duration=0).

### Effect size

We calculated a cell viability reduction caused by TMZ compared with the corresponding untreated control as raw mean difference with all data given in relation to the control viability as:

$$Cell\_viability\_reduction=D=\frac{Viability_{Con}-Viability_{TMZ}}{Viability_{Con}}$$

with its variance:

$$V_{D}=\frac{n_{Con}+n_{TMZ}}{n_{Con}\times n_{TMZ}}\times \frac{\left(n_{Con}-1\right){\left(\frac{{SD}_{Con}}{{Viability}_{Con}}\right)}^{2}+\left(n_{TMZ}-1\right){\left(\frac{{SD}_{TMZ}}{{Viability}_{Con}}\right)}^{2}}{n_{Con}+n_{TMZ}-2}$$

where n_Con_ and n_TMZ_ represented the number of experiments in the control and TMZ group, respectively, and SD_Con_ and SD_TMZ_ represented the SD of cell viabilities in the control and TMZ group. If the variance in the control group was not reported, we assumed this to be equivalent to variance in the corresponding TMZ group. If it was not clear whether variance was reported as SD or SEM, we assumed they were SEM, as a more conservative approach.

### Multilevel random-effects meta-analysis

We used random-effects meta-analysis.^21^ Because a single article might contribute several effects sizes, we used a three-level model, where the first level represented the raw cell viability data, the second level represented all the effects from a given article and the third level represented the article itself. This accounts for the relative non-independence of effects reported in the same article.^22^ Moreover, as the exact correlations of the dependent effects within an article were unknown, we used robust variance estimation.^23^

To estimate τ^2^, we used the restricted-maximum-likelihood method.^24^ This method has recently been shown to be robust for non-normally distributed effect sizes^25^ and is recommended for the estimation of τ^2^.^25 26^ We used the t-distribution for the calculation of the weighted mean effect as this accounts for uncertainty in the estimation of τ^2^.^27^ We took the within-level-three estimate of τ^2^ as a measure of reproducibility of findings between studies and subsequently as an indicator of irreproducibility of results across articles.

### Meta-regression

We tested 10 a priori defined parameters of which we expected reproducibility moderation (plus article reporting, TMZ concentration and treatment duration) in univariable meta-regressions. For these, a reduction of within-level-three τ^2^ would indicate that the parameter moderated reproducibility, with lower within-level-three τ^2^ indicating that findings would be more likely to reproduce if that parameter was controlled between the original and replicating experiments. We took the same approach to establish any effect of articles overall reporting quality or JIF. We also used univariable meta-regression to analyse the effects of TMZ dose and treatment duration. We transformed TMZ concentrations into a four-parameter log-logistic dose–response model.^28^ A similar four-parameter log-logistic time–response model was used for the treatment durations. Finally, we conducted multivariable meta-regression of the effect of dose, duration and moderators proven significant in univariable meta-regression. In all analysis, we set a significance level of 0.05.

### Analysis of reporting of published work since the time point of systematic search

Due to the relatively long interval between systematic literature search and publication of this review, we conducted an updated literature search on 10 March 2022 to investigate whether reporting quality may have changed during this period. Thereby, we assessed the study parameter reporting and the prevalence of potential risks of bias in the identified additional articles. The same rules for literature screening and data collection were applied as described above. Changes in overall reporting quality and risks of bias prevalence were tested with the Mann-Whitney-Wilcoxon test.

### Software

To remove duplicate articles from the systematic search results, we used two approaches, the deduplication function integrated in Zotero^29^ and one developed by the CAMARADES group.^30^ Articles were removed if they were detected as a duplicate by both functions. Afterwards, articles were manually screened for remaining duplicates. Screening and data extraction used the Systematic Review Facility for preclinical systematic reviews.^31^ Graphically presented data were extracted with the WebPlotDigitizer.^32^ Meta-analysis was performed within the RStudio environment^33^ using the programming language R.^34^ We used the rma.mv function of the R package Metafor^35^ for multilevel meta-regressions, the R package clubSandwich^36^ for robust variance estimation, the R package orchard^37^ for the calculation of marginal R^2^ and I^2^, the drm function of the R package drc^38^ for the dose–response and time–response models and the lm function of the integrated R package stats^34^ for linear regressions. The full statistical code and its datasets are publicly available on the GitHub data repository.^39^

## Results

### Systematic search

**Figure 1:**
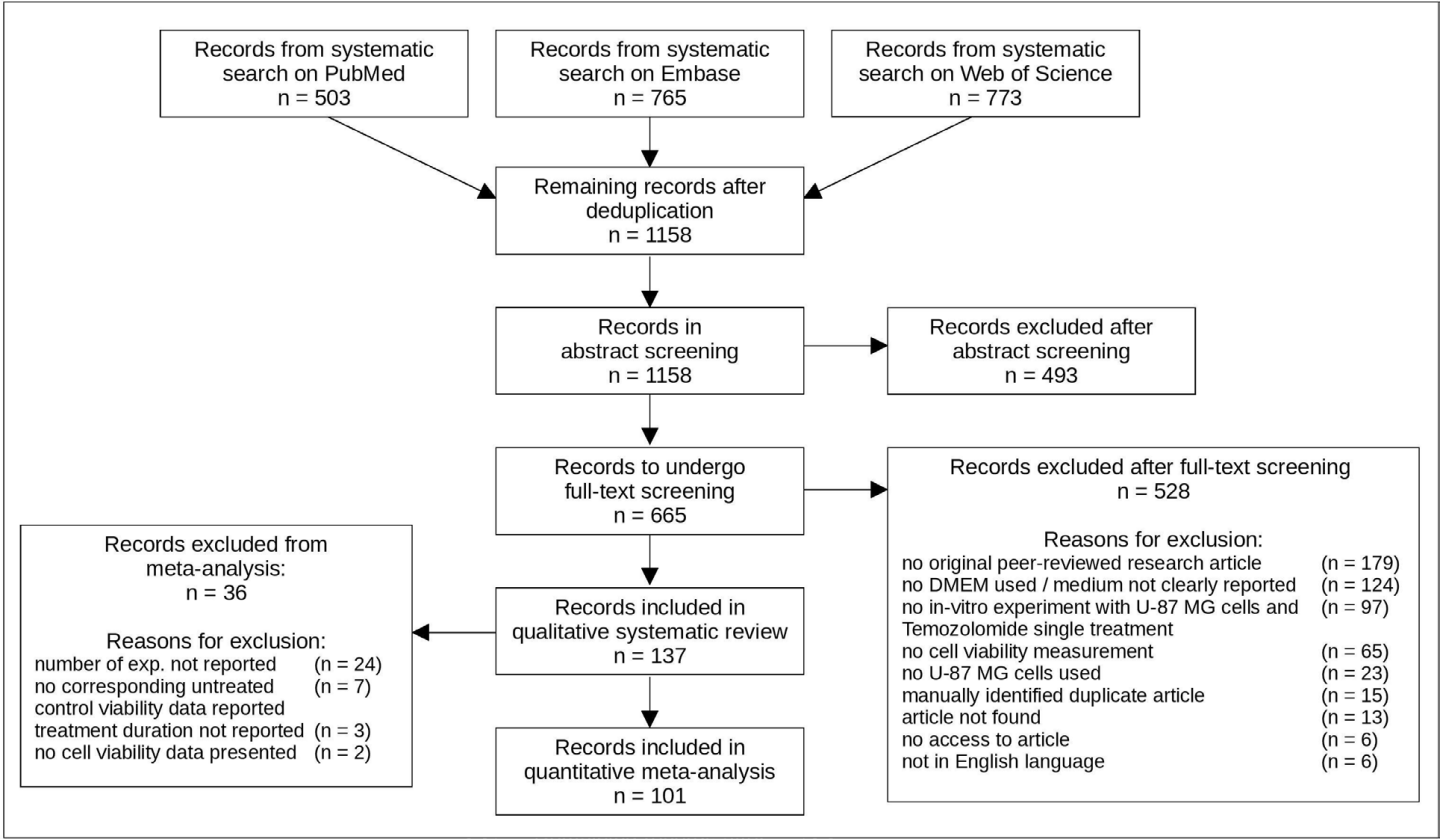
Systematic search and screening results. Presentation based on the PRISMA statement.^68^ Systematic search was conducted in August 2020. Qualitative analysis included all calculations in this review except meta-analysis and meta-regressions. One reason for exclusion per excluded article. DMEM, Dulbecco’s Modified Eagle’s Medium; Exp, experiments; U-87 MG, Uppsala-87 Malignant Glioma.

We identified 1158 publications of which 137 articles met our inclusion criteria and were included in the systematic review; 101 provided sufficient data to be included in the meta-analysis (figure 1). Online supplemental additional data A6^19^ contains a list of all included articles. These 137 articles described 828 experiments, where every different combination of drug concentration and treatment duration used was considered as an individual experiment. The main reason for exclusion from meta-analysis was an unreported number of contributing experimental units (n=24 articles).

### Experimental parameters distribution

Across 137 publications, a broad range of experimental characteristics was observed. The most common source of U-87 MG cells was the ATCC (66 articles, 48.2% of all included articles; table 1). A cell line authentication report was available in 16 articles (11.7%), and 8 articles (5.8%) described testing for mycoplasma contamination. The reported cell passage number ranged from 3 to 100 (median of 15), but 123 publications (89.8%) did not report it. Only 29 of 137 articles (21.2%) reported the level of glucose in culture medium, and in these high glucose supplementation (4500 mg/dL) was most prevalent (in 24 of 29). Control treatment was dimethyl sulfoxide (DMSO) in 37 articles and culture medium alone in 13 articles; in 87 articles, it was not reported or only labelled as ‘untreated control’ without further specification. The most common cell viability assessment method was the 3-(4,5-dimethylthiazol-2-yl)-2,5-diphenyltetrazolium bromide (MTT) assay (67 articles, 48.9%). The concentration of U-87 MG cells used ranged from 5 cells/µL to 500 cells/µL (table 2), with a median of 30 cells/µL. Ninety-three articles included information for the number of cells per well but not the volume in which these cells were plated. Cells were passaged based on confluence (13 articles, ranging from 50% to 90% confluence) or on time (4 articles, ranging from 2 days to 7 days), but criteria for cell passaging were not stated in 120 studies (87.6%; table 3).

**Table 1:** Extracted parameters

Parameter	Phenotype	Articles	
**General article information**			
Conflict of interests statement	Declaration of no conflict of interests	86	62.8%
	Declaration of existing conflict of interests	5	3.6%
	No statement about conflict of interests	46	33.6%
**U-87 MG in vitro model**			
Source of U-87 MG cells	ATCC, Manassas, Virginia, USA	66	48.2%
	Chinese Academy of Sciences, Beijing, China	27	19.7%
	Other commercial/institutional sources	24	17.5%
	Colleagues	11	8.0%
	Not reported	9	6.6%
U-87 MG cell line authentication conducted	Yes	16	11.7%
	No/not reported	121	88.3%
U-87 MG age (maximum number of cell passage)	3	1	0.7%
	7	1	0.7%
	8	1	0.7%
	10	3	2.2%
	15	4	2.9%
	20	2	1.5%
	35	1	0.7%
	100	1	0.7%
	Not reported	123	89.8%
**U-87 MG culture conditions**			
Glucose level of cell culture medium	Low glucose (1000 mg/dL)	3	2.2%
	High glucose (4500 mg/dL)	24	16.8%
	Low and high glucose (in different experiments)	1	0.7%
	Without glucose	1	0.7%
	Not reported	108	78.8%
Mycoplasma contamination checked	Yes	8	5.8%
	Not reported	129	94.2%
Supplemented antibiotics	Penicillin and streptomycin	92	67.2%
	Other antibiotics	5	3.6%
	No antibiotics supplemented	3	2.2%
	Not reported	37	27.0%
Source of FBS	Thermo Fisher Scientific, Waltham, Massachusetts, USA (including Gibco, Invitrogen and Life Technologies)	51	37.2%
	Hyclone Laboratories, Logan, Utah, USA	13	9.5%
	Sigma-Aldrich, St Louis, Missouri, USA	8	5.8%
	Other sources	22	16.1%
	FBS was not used	1	0.7%
	Not reported	42	30.7%
**Control group and outcome measurement**			
Type of untreated control	Drug vehicle (DMSO)	37	27.0%
	Cell culture medium only	13	9.5%
	Not reported	87	63.5%
Cell viability assessment method	MTT assay, colorimetric	67	48.9%
	CCK8, colorimetric	20	14.6%
	SRB assay, colorimetric	9	6.6%
	Alamar Blue assay, colorimetric	7	5.1%
	Trypan Blue Exclusion test, cell counting	6	4.4%
	WST-1 assay, colorimetric	6	4.4%
	MTS assay, colorimetric	3	2.2%
	Other assessment methods	11	8.0%
	More than one assay used	8	5.8%

Extracted parameter phenotypes (including additional information obtained through contacting the authors). One phenotype per article. The column ‘Articles’ shows the absolute and relative frequencies of articles with the parameter phenotype in relation to all 137 included articles.

ATCC, American Type Culture Collection; CCK8, Cell Counting Kit-8; DMEM, Dulbecco’s Modified Eagle’s Medium; DMSO, dimethyl sulfoxide; FBS, fetal bovine serum; U-87 MG, Uppsala-87 Malignant Glioma; MTS, 3-(4,5-dimethylthiazol-2-yl)-5-(3-carboxymethoxyphenyl)-2-(4-sulfophenyl)-2H-tetrazolium; MTT, 3-(4,5-dimethylthiazol-2-yl)-2,5-diphenyltetrazolium bromide; SRB, Sulforhodamine B; WST-1, water soluble tetrazolium 1.

**Table 2:** Extracted cell concentrations

Cell concentration (cells/µL)	Articles	
5	1	0.7%
12.5–62.5	1	0.7%
15	1	0.7%
20	3	2.2%
25	3	2.2%
30	4	2.9%
40	2	1.5%
50	5	3.6%
100	1	0.7%
166.7	1	0.7%
200	1	0.7%
500	1	0.7%
Reporting of only the number of cells per well without the associated volume per well	93	67.9%
No information regarding the cell number, the volume they are plated in or the cell concentration was given	20	14.6%

Extracted cell concentrations (including additional information obtained through contacting the authors). One concentration per article. The column ‘Articles’ shows the absolute and relative frequencies of articles with the parameter phenotype in relation to all 137 included articles.

**Table 3:** Extracted cell passaging criteria

Criterion	Articles	
Based on cell culture confluence		
50%–70%	1	0.7%
60%–80%	1	0.7%
70%	2	1.5%
70%–90%	3	2.2%
80%	6	4.4%
Based on time intervals		
2 days	1	0.7%
2–3 days	1	0.7%
3–4 days	1	0.7%
7 days	1	0.7%
No cell passaging criteria were reported	120	87.6%

Extracted cell passaging criteria (including additional information obtained through contacting the authors). One criterion per article. The column ‘Articles’ shows the absolute and relative frequencies of articles with the parameter phenotype in relation to all 137 included articles.

Overall, 98 different TMZ concentrations (10nM–16.0 mM; median: 100 µM) and 20 different treatment durations (4 hours–12 days; median: 3 days) were reported, as illustrated in online supplemental additional data A7 and A8, respectively. In several articles, it was not clear whether cell viability was measured directly after TMZ exposure, or whether there was a ‘wash out’ or recovery period. In others, it was unclear whether TMZ was added to the cells once and remained in suspension or whether TMZ was added repeatedly at different times. For the purposes of the meta-analysis, we assumed a single TMZ addition with continuous incubation for the reported time followed directly by the assessment of cell viability.

### Completeness of reporting

**Figure 2:**
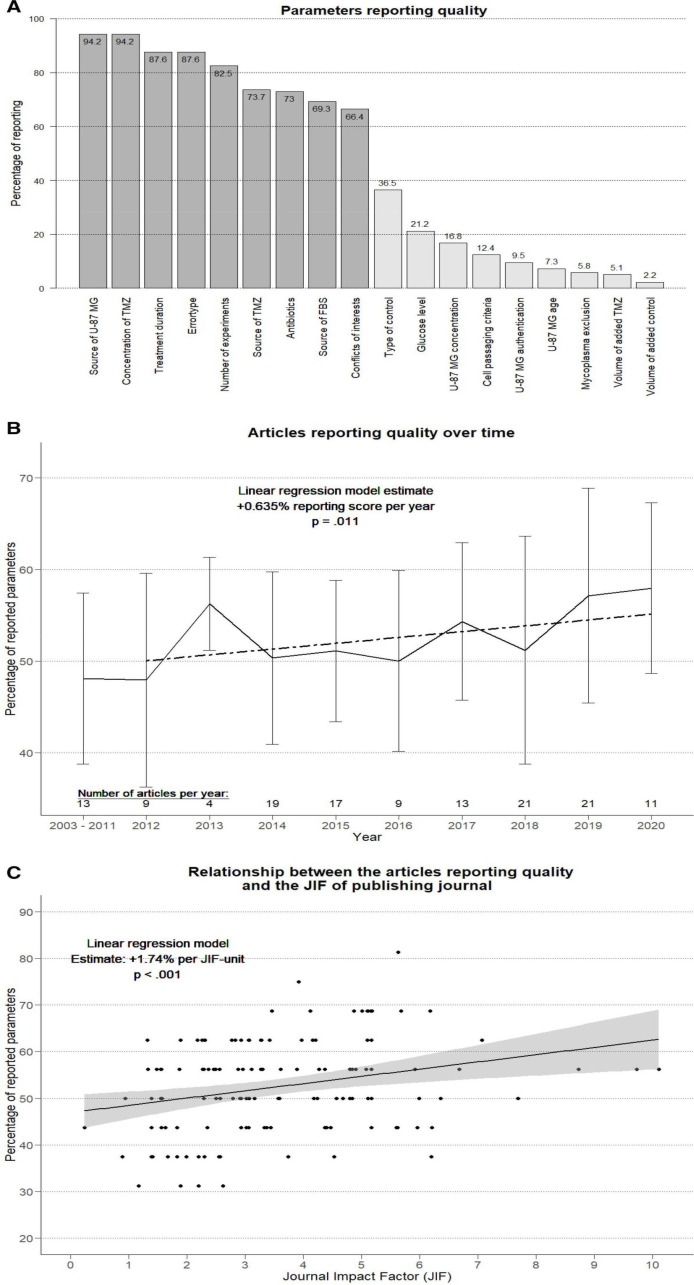
Reporting quality (of parameters, over time and depending on the JIF). (A) The reporting quality of a parameter was defined as the share of articles that reported this parameter in comparison to all 137 included articles. This share is shown on the top of each bar for each parameter. A parameter was considered to be reported if its phenotype was clear based on the information provided in the original full-text research article. (B) Linear regression model of articles reporting quality (proportion of reported parameters to 16 experimental parameters) and year of publication. The articles published before 2012 were graphically summarised because of low numbers of articles published in these years (but the exact years were used for the regression). Only articles published until the time of systematic search in August 2020 were considered. The dotted line represents the linear regression prediction; error bars indicate the SD around the mean reporting score per year represented by the continuous line. (C) Linear regression model of articles reporting quality and the JIF of the articles publishing journal in the year of publication. The grey area marks the 95% CI of the regression model prediction. JIFs were obtained from the Clarivates Incites Journal Citation Reports (Web of Science group, 2020). For the articles published in 2020, the JIF of 2019 replaced the JIF of 2020 as the more recent was not available at the time of analysis. One article was omitted as no JIF could be obtained. FBS, fetal bovine serum; JIF, journal impact factor; TMZ, temozolomide; U-87 MG, Uppsala 87 Malignant Glioma.

Several key experimental parameters were reported in fewer than half of the articles: The type of untreated control was reported in 36.5%, the glucose level of culture mediums in 21.2% and U-87 MG cell age in 7.3% of all 137 articles (figure 2A). The median number of quality items reported was 8.4 of 16 (ranging from 3 to 13). Analysis of change over time suggested slight reporting improvement (+0.635% parameter reporting ratio per article per year, p=0.011; figure 2B); and reporting quality seemed to be higher for articles published in journals with higher impact factors (+1.74% per unit increase in JIF unit, p<0.001; figure 2C).

### Reporting of measures to reduce risks of bias

Not one of 137 articles described a sample size calculation, random allocation to experimental group, blinded outcome assessment or the use of a preregistered protocol specifying the hypotheses and outcomes as shown in the online supplemental additional data A9.^19^ The methods used to calculate cell viability average and error values were unclear in 92 articles, and the number of independent experiments and technical replicates per experiment conducted were unclear in 47 articles. The mean number of measures to reduce risks of bias reported was 2.9.

### Reporting and risks of bias in publications published after systematic search conduction

In the interval between the initial systematic literature search (August 2020) and March 2022, the literature flow chart shown in online supplemental additional data A10^19^ presents 41 additionally published articles. As shown in online supplemental additional data A11,^19^ most experimental parameters offered negligible variations between the time periods, including the glucose level of cell culture medium (−1.7% less reported compared with initial search). The largest improvements comprised conflicts of interests (+26.3 %) and cell concentration reporting (+17.3 %). One notable deterioration was less frequent explicit reporting of independent experiments and their technical replicates (−21.8 %), resulting in a lower rate of unambiguous computational pathways for cell viability mean and error values (−13.3 %). However, the only significant change was observed in an improved conflicts of interests reporting. A detailed comparison of reporting and risks of bias is given in the online supplemental additional data A12.^19^ Meanwhile, the overall articles reporting quality and prevalence of risks of bias has not changed significantly as indicated in online supplemental additional data A13 and A14^19^ (p=0.390 and p=0.751, respectively (Mann-Whitney-Wilcoxon test)).

### Meta-analysis of the effect of TMZ

The observed effect of TMZ is highly heterogeneous; variation within different experiments in the same article (represented by I^2^ of level-two variance) accounts for 56.6% of observed variance, variation of the effect across different articles (represented by I^2^ of level-three variance) accounts for 42.9% of observed variance, and the variance due to random chance expected if all experiments of all articles were held under identical conditions made up only 0.5% of the total variance (table 4). The heterogeneity of results across the articles is reflected in an SD (τ) of ±16.6% (for this SD estimate, 95% CI 13.9% to 19.8%) around a global estimate of a reduction in cell viability following TMZ treatment of 33.8% (95% CI 30.0% to 37.7%) compared to the untreated control.

**Table 4:** Random-effects three-level meta-analysis suggests significant irreproducibility

Meta-analysis data	
Number of included effects of TMZ on U-87 MG viability	644
Number of included articles the effects were reported in	101
**Effect of TMZ**	
Overall weighted mean effect of TMZ (U-87 MG viability reduction compared to untreated control)	33.8% (30.0% to 37.7%)
**Investigation of heterogeneity**	
Test whether heterogeneity is present	
Q (df=643)	134 066.1
P value	<0.001
Total I^2^	99.5%
Within-articles variance of the true effect	
τ^2^	3.6% (3.2% to 4.1%)
τ	19.0% (17.9% to 20.3%)
I^2^ (=proportion of total variance)	56.6%
Between-articles variance of the true effect (~irreproducibility)	
τ^2^	2.8% (1.9% to 3.9%)
τ	16.6% (13.9% to 19.8%)
I^2^ (=proportion of total variance)	42.9%

Random-effects three-level meta-analysis using the raw data the effects were calculated with as first level, the effect sizes within each article as second level and the articles the effects were reported in as third level. τ^2^: estimator of the variance of true effects (level-two variance: within-articles variance; level-three variance: between-articles variance: representant of irreproducibility); τ: square root of τ^2^; I^2^: proportion of within-articles and between-articles variance, respectively, of the total observed variance, including sampling error. τ^2^ estimator: restricted-maximum likelihood. Cochran’s Q was used as the test for heterogeneity using a χ^2^ distribution; values in parenthesis show CIs with significance level set at 0.05.

df, degree of freedom; U-87 MG, Uppsala-87 Malignant Glioma; TMZ, temozolomide.

### Drivers of heterogeneity

The within-articles variance of effects reported in the same article was, as expected, partly explained by differences in TMZ concentrations (48.3%) and treatment durations (4.8%) and is presented in the online supplemental additional data A15.^19^ However, both features did not explain parts of between-articles variance of effects (table 5). Combining TMZ concentration and treatment duration in a multivariable meta-regression reduced within-articles variance τ^2^ from 3.6% to 1.7%, while the estimated between-articles variance τ^2^ increased from 2.8% to 3.7% (table 6).

**Table 5:** Moderators of between-articles variance of true effects

Moderator	Type	Number of effects	Number of articles	P value	Marginal R^2^	Between-articles variance		
						τ^2^	I^2^	Explained
Without moderators		644	101			2.8%	42.9%	n.a.
U-87 MG source	cat.	644	101	0.075	n.s.	2.6%		
U-87 MG authentication	cat.	644	101	0.476	n.s.	2.8%		
U-87 MG age (cell passages)	cont.	138	11	0.238	n.s.	1.5%		
Cell concentration	cont.	113	20	0.323	n.s.	3.8%		
Confluence level at cell passaging	cont.	57	11	0.319	n.s.	0.8%		
Glucose level of culture medium	cat.	644	101	0.016	7.0%	2.5%*	40.1%	10.9%
Mycoplasma exclusion	cat.	644	101	0.491	n.s.	2.8%		
Supplemented antibiotics	cat.	644	101	0.094	n.s.	2.6%		
FBS source	cat.	644	101	0.067	n.s.	2.6%		
Type of untreated control	cat.	644	101	0.370	n.s.	2.8%		
Articles reporting quality	int.	644	101	0.031	3.3%	2.6%†	41.7%	5.0%
TMZ conc.	cont.	644	101	<0.001	38.6%	3.4%‡	64.3%	0.0%
Treatment duration	cont.	644	101	<0.001	6.0%	2.9%§	45.7%	0.0%

Random-effects three-level meta-regressions with the raw data the effects were calculated with as first level, the reported effects as second level and the articles the effects were reported in as third level. Marginal R^2^ indicates the regression model fit^69^; τ^2^: estimator of the variance of true effects; τ: square root of τ^2^; I^2^: proportion of between-articles variance of the total observed variance, including sampling error. τ^2^ estimator: restricted-maximum likelihood. The column ‘Explained’ indicates the reduction of τ^2^ after including the particular moderator compared with τ^2^ without moderators (only applicable if the number of included effects and articles is identical). For some continuous moderators, the number of effects and articles included in the regression is reduced due to non-reporting, which leads to a limited comparability of τ^2^ between parameters with different numbers of belonging articles and effects. ‘Not reported’ was included as a category for categorical moderators. P value is given for the test of the moderator. R^2^, I^2^ and the explained heterogeneity were only calculated for moderators that prove significance in the test of the moderator (α=0.05).

*95% CI of τ^2^: 1.7% to 3.6%.

†95% CI of τ^2^: 1.8% to 3.8%.

‡95% CI of τ^2^: 2.5% to 4.8%.

§95% CI of τ^2^: 2.1% to 4.2%.

cat., categorical; cont., continuous; FBS, fetal bovine serum; int., interval; n.a., not applicable; n.s., not significant; TMZ conc., temozolomide concentration; U-87 MG, Uppsala-87 Malignant Glioma.

**Table 6:** Multivariable meta-regressions

Moderators	P value	Marginal R^2^	Within-articles variance		Between-articles variance	
			τ^2^	Adjusted I^2^	τ^2^	Adjusted I^2^
Without moderators			3.6%	56.6%	2.8%	42.9%
TMZ concentration and treatment duration	<0.001	42.1%	1.7% (1.5% to 1.9%)	30.9%	3.7% (2.7% to 5.2%)	68.5%
TMZ concentration, treatment duration and mediums’ glucose level	<0.001	45.4%	1.7% (1.5% to 1.9%)	31.9%	3.5% (2.6% to 5.0%)	67.4%
TMZ concentration, treatment duration and articles reporting quality	<0.001	44.1%	1.7% (1.5% to 1.9%)	31.6%	3.6% (2.6% to 5.0%)	67.8%
TMZ concentration, treatment duration, mediums’ glucose level and articles reporting quality	<0.001	45.9%	1.7% (1.5% to 1.9%)	32.0%	3.5% (2.5% to 5.0%)	67.4%

Multivariable random-effects three-level meta-regressions with the raw data the effects were calculated with as first level, the reported effects as second level and the articles the effects were reported in as third level. P value is given for the test of the moderator. Marginal R^2^ indicates the regression model fit;^69^ τ^2^: estimator of the variance of true effects; adjusted I^2^: proportion of within-articles and between-articles variance of true effects, respectively, of the total observed variance including sampling error with the indicated moderators. τ^2^ estimator: restricted-maximum likelihood. For all multivariable meta-regressions, 644 effects in 101 articles were included.

TMZ, temozolomide.

The glucose level in the cell culture medium was the only experimental parameter significantly associated with heterogeneity of results across the articles (p=0.016, table 5). The moderator fit indicated by marginal R^2^ was 7.0%, and 10.9% of the between-articles variance τ^2^ could be explained because of differences in the glucose level. In other words, roughly 11% of results variation was attributed to different glucose levels used in the culture medium. The different glucose levels and effects are shown in online supplemental additional data A16,^19^ indicating a smaller effect of TMZ in the articles using high glucose supplementation (cell viability reduction of 23.1% compared to the untreated control, 95% CI 15.8% to 30.5%) than in the articles using an unreported glucose level (37.1%, 95% CI 28.6% to 45.6%, p=0.002).

Articles reporting quality showed a significant correlation with the reported effect of TMZ (marginal R^2^=3.3%). Reported effect on cell viability fell by 3.0% for each unit increase in the number of reported parameters (p=0.026). Adding these two features (glucose level and articles reporting quality) to the multivariable between-articles variance meta-regression, including drug concentration and treatment duration, resulted in a slightly improved model, reducing τ^2^ from 3.7% to 3.5%.

## Discussion

### Reporting and experimental parameters

We found a highly significant relationship between the concentration of TMZ used, the duration of treatment and the measured effect of TMZ on the viability of U-87 MG cells (p<0.001). However, the reporting of experimental parameters in this literature—including such fundamental issues as the concentration of the drug and the treatment duration—is limited. A recent study has also identified suboptimal reporting of basic experimental parameters and varying cell viability reducing effects of TMZ in in vitro glioma cell line experiments with TMZ single treatment.^14^

Based on TMZ concentrations found in peritumoral tissue^40 41^ and cerebrospinal fluid,^42^ Stepanenko and Checkhonin recommended in vitro studies should use TMZ concentrations of 1–10 µM.^43^ Although the effect of higher concentrations could be examined, it seems reasonable to expect that publications use at least one clinically relevant drug concentration. More than two-thirds of articles (70 of 101 articles included in meta-analysis) did not use clinically relevant TMZ concentrations in at least one of their experiments. The effects of TMZ are due to DNA alkylation and methylation, and this requires TMZ internalisation, which usually occurs during cell division. An effect of TMZ can, therefore, be expected at the earliest after about 1.5 cell doubling times, and the cell doubling time of U-87 MG is around 34 hours.^44 45^ Applying an early credible limit for efficacy of 51 hours, 31.7% of the articles included in the meta-analysis only measured effects before they could reasonably be expected to occur. This could lead to an underestimation of the effect of TMZ, or an overestimated effect of new drug candidates compared with TMZ, if the new drug candidates have an earlier onset of their effect.

Limited reporting of key statistical properties such as the number of experimental units (not reported in 24 of 137 articles) or the type of error presented in results (not reported in 17 articles) is of concern, and we note recommendations for improved reporting of such items.^46^

To introduce sufficient independence between repetitions of cell culture experiments, it has been suggested that each experiment should be conducted on a different day, with freshly prepared materials, and that the experimental unit defined as the day, so n is taken as the number of days.^47 48^ Along with the limited reporting of the number of independent experiments and (technical) replications per experiment, this leads us to encourage researchers to clearly describe their methods for introducing robust independence.

Despite the known problems with the provenance of this cell line^17^ and the widely recommended implementation of cell line authentication,^49 50^ we were surprised that the great majority (90.5%) of included articles did not report such an identification procedure. In addition, infrequent reporting of U-87 MG cell passage number and cell concentration used in the model adds to concern that published in vitro glioma research may be particularly confounded, as it is known that both parameters are potent drivers for heterogeneity.^51 52^ Importantly, our analysis identified an encouraging but slow trend of improving reporting over time. This is in line with recent findings of a large study showing that methodological reporting quality for 1 578 964 PubMed Central articles had increased in recent times.^53^ Interestingly, although showing statistical significance, the positive correlation of elevated impact factor of the journals the articles are published in and reporting quality was limited, again consistent with the findings of Menke *et al*. We note also that the anti-cell growth effect of TMZ was greater in publications which had less complete reporting of experimental details, consistent with previous work on in vivo studies which showed a higher effect of a therapeutic intervention reported in articles with worse methodological reporting and higher risks of bias.^54^

While rigid compliance to reporting guidelines may be seen by some as unduly burdensome, our findings suggest that adoption of guidelines for the design, conduct, analysis and reporting of in vitro research such as the materials design analysis reporting (MDAR) framework^46^ would lead to a more valuable in vitro research.

### Sources and amplitudes of heterogeneity

The observed heterogeneity was far in excess of that expected from random sampling error (p<0.001), even though we had taken steps to include broadly similar studies (outcome measurement and culture medium). As the mean effect estimate of TMZ—across all articles with all applied drug concentrations and treatment durations—is a cell viability reduction of 33.8% compared with the untreated control, the magnitude of the SD of the effects across the articles with ±16.6% is almost half as high as the effect estimate itself. These strongly heterogeneous findings are in line with earlier quantifications of results repeatability in cancer research^2 8^ and with the ‘reproducibility project: cancer biology’.^55^ These investigations evaluated reproducibility in a one-on-one replication attempt and calculated the share of reproducible articles as the measure of reproducibility in a field. In contrast, our meta-analysis retrospectively extracted every published effect of a commonly performed experiment and calculated the variance of the effects across the articles as a measure of irreproducibility. We believe that this strategy holds an advantage in that it enables us to recognise which study parameters may act as drivers of irreproducibility. Furthermore, we think our approach is closer to scientific reality, since most studies are carried out relatively independent of each other and are not the focus of one-to-one replication of selected previously published results.

The effect of TMZ was almost 40% lower in the high glucose group than in the articles with an unreported glucose level. It is known that glucose restriction leads to a reduced cell proliferation^56^ and to a sensitisation of glioblastoma cells to TMZ,^57 58^ so we consider it likely that the articles with unreported glucose levels mainly used low glucose conditions. However, as only two articles included in the meta-analysis reported the use of low glucose levels, we were not able to obtain precise enough estimates for the effect in the low glucose group. For the other experimental parameters tested in meta-regressions, no significant moderation was observed. The results do not demonstrate that there was no moderation of reproducibility and may simply reflect the statistical power of our approach depending on a minimum frequency of reporting of a particular parameter tested for its impact on reproducibility. For example, only 11 studies could be included in the meta-regression analysing the relevance of different levels of cell culture confluence at time of cell passaging, and we acknowledge this is a limitation of our study.

### Implications for future research

Despite the existence of in vitro cell culture experimental guidelines like the Guidance on Good Cell Culture Practice,^52^ there are no widely applied reporting guidelines specific to in vitro preclinical research, although the MDAR framework includes in vitro research. Randomised group allocation, blinded outcome assessment and sample size calculation are well-established methods to reduce risks of bias in clinical and in vivo research, but in this review none of the included articles reported any one of these methods. This is consistent with previous findings,^59 60^ and some have argued for the implementation of randomisation and blinding in in vitro trials.^2 61^ Care would be required to mitigate any additional risks due to pipetting errors (because of more complex pipetting schemes) and more challenging data transfers,^52^ but the risk of unconscious systematic bias in cell plating and pipetting on multiwell plates might decrease.^62^ Although, we recommend random allocation of wells to experimental groups, blinding of cell culture procedures and assessment of cell viability to reduce potential bias. Meaningful sample size calculations will require better understanding of the experimental unit, and we endorse the suggestion that sufficient independence between replicates should be introduced by performing experiments on different days with freshly prepared cells and reagents.^47 48 63^

The choice of control in cell culture drug response assays is important, and we were concerned that the exact condition of the untreated control arm was not reported in the majority of cases. Of note, caution is advised in the selection of maximum volume percentages of common control treatments, that is, DMSO, as elevated dosage causes inhibition of cell proliferation, exposing risk for efficacy normalisation of an investigated intervention.^52^ Where TMZ had been used as an active control for the evaluation of new therapeutic candidates, the parameters for the new drug were often much more detailed described than those for the control treatment with TMZ. As an example of why this might be important, if the effects of different drugs are differentially sensitive to glucose levels, this may lead to erroneous interpretations of the potency of a new investigational drug.

### Limitations

Our study has several limitations. We did not choose the parameters contributing to the reporting quality based on a pre-existing reporting guideline because we could not identify an appropriate guideline for this type of experiment. Instead, we used parameters derived from previous work and from laboratory experience. It is unlikely that the chosen parameters have the same impact on heterogeneity, but we had no basis to assert their different impact, and so used an unweighted score.

The overwhelming part of heterogeneity of results across the articles (89.1%) remained unexplained. As discussed earlier, the main limiting factor during the analysis was the surprisingly low frequency of reporting of experimental parameters of interest, which will have limited the power of our analysis. Further reasons for unexplained heterogeneity include contributions to irreproducibility by parameters not included in the review, or different behaviours of U-87 MG cells in different laboratories for reasons unrelated to study design.

Although our study included analysis on reporting of the source, authentication and cell passage, we are aware that a larger analysis with multiple different cancer cell models would be required to draw firmer conclusions. We think that the similar use of other glioblastoma cell lines means that our findings are probably transferable to these models.

Since the initial literature search was conducted in August 2020, we implemented an updated systematic search conducted in March 2022 and identified—besides more frequent conflicts of interest disclosures—no substantial changes in the methodological reporting habits in in vitro glioma research. This indicates that our findings reflect the contemporary practice in this scientific field, probably beyond the lines of neuro-oncologic research and may also provide a baseline for further projects assessing the reporting standards and reproducibility in different aspects of (in vitro) cancer research. Importantly, the reporting of glucose level in cell culture medium has not improved, highlighting the actuality of this review. The raise in reporting conflicts of interest may have been the result of a wider application of updated conflicts of interest disclosure guidelines by the International Committee on Medicine Journals Editors.^64^

Finally, we had planned to conduct a parallel review of research using more contemporary glioblastoma in vitro models, such as 3D glioma stem-like cells (GSC) approaches (eg, NCH421k^65^). However, in preliminary searches, we found that there was no commonly used GSC model, with authors generally using individually generated cell lines. It seemed not reasonable to perform a meta-analysis on these limited data. However, as these GSC models are probably better representatives of disease pathophysiology,^66 67^ accurate and comprehensive reporting on experimental parameters may be even more important to ensure reproducibility of their results, as elevated genetic and cellular complexity of these cells may translate into larger intrinsic biological variations.

## Conclusion

In vitro glioma research suffers from insufficient reporting of methods and experimental design. We believe that current publication practices contribute as one source of variance that may be a driver for poor reproducibility. Although our analysis contrasts current practice with an idealised scenario and must be considered with caution in some regards (ie, risks of bias), our study clearly supports the establishment of consensus reporting guidelines for in vitro (cancer) research. Our review should be considered as an independent confirmatory study of earlier reporting and reproducibility enhancing recommendations^52^ with the additional benefits of evidence for insufficient methodological reporting as well as quantification of the reproducibility of results in a highly relevant area of in vitro brain cancer research. It may be relevant for raising further awareness in a wider audience of stakeholders in biomedical research.

## Data Availability

Data are available in a public, open access repository. The statistical code and its datasets (initial systematic search and updated search) are publicly available on the GitHub data repository (https://github.com/TimoSander/Reporting-practices-as-a-source-of-heterogeneity-in-in-vitro-cancer-research, License: CC-By Attribution 4.0 International). Moreover, additional data to the manuscript (including supporting figures, tables, and detailed updated search results) are permanently publicly available at the Open Science Framework (https://osf.io/knhx8/, DOI: 10.17605/OSF.IO/KNHX8, License: CC-By Attribution 4.0 International). The data are readily accessible and have no limitations for viewing. In addition, an ex-ante study protocol specifying methods and objectives of the review is also available at the Open Science Framework (https://osf.io/u6yhb/, DOI: 10.17605/OSF.IO/U6YHB, License: CC-By Attribution 4.0 International).
