# Supplementary material for: Meta-analysis on reporting practices as a source of heterogeneity in in vitro cancer research
Source: BMJ Open Sci. 2022 Jun 1;6(1):e100272. doi: 10.1136/bmjos-2021-100272 (PMC9171230; doi:10.1136/bmjos-2021-100272)
Supplement: Supplementary data [file bmjos-2021-100272supp001.pdf]

Additional data to *Meta-analysis on reporting practices as a source of heterogeneity in in vitro cancer research*

A1: Exact search strategy

(glioblastom OR Astrocytom*, Grade IV OR Glioblastom* Multiform* OR Giant Cell Glioblastom* OR Brain cance* OR Malignant Gliom*) AND (Temozolomide OR Temodal OR Temodar OR methazolastone OR tmz) AND (U87 OR U-87 OR U87MG OR U87-MG OR U 87-MG OR U-87-MG OR U87 GBM OR U87-GBM OR U 87 GBM OR U-87 GBM OR U-87-GBM)*

A2: Literature screening criteria

Inclusion criteria	Exclusion criteria
U-87 MG cell line as glioblastoma *in vitro* model	Other models than U-87 MG cell line<br>*In vivo* models, Xenotransplantation models
TMZ single treatment	TMZ as a part of a combined treatment with other drugs or genetic interventions
Comparison of the effect of TMZ to an untreated control	No comparison to an untreated control
Cell viability assessment (MTT and similar colorimetric assays, cell counting) to quantify the effect of TMZ	Effect of TMZ measured with none of these cell viability assessment methods
DMEM as the cell culture medium	Other cell culture media than DMEM
Original peer-reviewed research articles	Other publication types (e.g., conference abstracts, poster presentations)
English language	Other languages than English

Articles were included if they met all inclusion criteria and no exclusion criteria. If an article included multiple experiments where one or more experiment did not match the criteria but at least one did match, then the article was included. DMEM = Dulbecco's Modified Eagle Medium; MTT = 3-(4,5-dimethylthiazol-2-yl)-2,5-diphenyltetrazolium bromide; TMZ = temozolomide; U-87 MG = Uppsala-87 Malignant Glioma.

A3: Extracted parameters from the included articles

Parameter	Possible phenotypes
**General article information**	
Title, Authors, Year, Publishing journals JIF	n. a.
Conflicts of interest's statement	Conflicts / no conflicts
**Cell model**	
U-87 MG cell line source	Name and country of source
U-87 MG cell line authentication reporting	Reported / not reported
U-87 MG cell line age reporting	Reported / not reported
**Cell culture conditions**	
Number/Volume/Concentration of U-87 MG cells	Exact Number/Volume/Concentration
Cell passaging criterion	Confluency/Time intervals
Glucose level of cell culture medium	Exact glucose concentration / high glucose / low glucose / no glucose
Reporting of successful mycoplasma exclusion test	Reported / not reported
Supplemented antibiotics	Name and dose of the antibiotics
FBS use and source	FBS supplemented / FBS not supplemented
	Name and country of source
**Intervention & control group**	
TMZ source	Name and country of source
Volume of the added TMZ suspension	Exact volume
Type of untreated control	Drug vehicle / medium only / other
Volume of the added control suspension	Exact volume
**Outcome**	
Cell viability of U-87 MG cells after incubation with TMZ and the corresponding untreated control	Mean and error data of cell viabilities / growth inhibition rates / proliferation rates
Concentration of TMZ	Exact concentration
Treatment duration	Exact duration
Number of experiments	Exact number of experiments
Type of outcome measurement assay	Name of assay

FBS = Fetal bovine serum; JIF = Journal Impact Factor (for the year of publication; obtained from Clarivates InCites Journal Citation Reports); U-87 MG = Uppsala-87 Malignant Glioma; TMZ = Temozolomide.

A4: Assessed potential risks of bias parameters

Were data for every relevant experiment mentioned in the methods section of a particular article presented?
Were data for every concentration of temozolomide and treatment duration presented?
Were the number of experiments and the number of replicates per experiment clearly reported?
Was a sample size calculation for the needed number of experiments reported?
Was the way calculating the U87-MG cell viability mean and error data clearly reported?
Was it reported whether the authors allocated the U87-MG cells randomly to the treatment and control group?
Was it reported whether the authors measured the U87-MG cell viability blinded?
Was a pre-registered study protocol available?

U-87 MG = Uppsala-87 Malignant Glioma.

A5: Parameters the authors were asked for if they were not reported in the article (and additional potential risks of bias parameters)

Missing information in the articles:
U-87 MG cell line authentication
U-87 MG age (maximum number of passages)
Glucose level of cell culture medium
Type of untreated control
Concentration of TMZ
Number of experiments and replicates per experiment
Type of error of the presented data (SD or SEM)
Treatment duration
Additional risks of bias parameters:
Therapy regime (single dose, multi dose or different)
Was the cell viability measured directly after the treatment of U-87 MG cells with TMZ?
Way of calculation of cell viability data (mean of all data, mean of means or different)

SD = standard deviation; SEM = standard error of the mean; U-87 MG = Uppsala-87 Malignant Glioma; TMZ = Temozolomide.

A6: Full list of included articles into the systematic review and meta-analysis

Article number	Title	Authors	Year	DOI	Included in meta-analysis
1	Afatinib and Temozolomide combination inhibits tumorigenesis by targeting EGFRvIII-cMet signaling in glioblastoma cells	Vengoji et al.	2019	10.1186/s13046-019-1264-2	TRUE
2	Akt and beta-catenin contribute to TMZ resistance and EMT of MGMT negative malignant glioma cell line	Yi et al.	2016	10.1016/j.jns.2016.05.054	TRUE
3	Anti-tumor activities of luteolin and silibinin in glioblastoma cells: overexpression of miR-7-1-3p augmented luteolin and silibinin to inhibit autophagy and induce apoptosis in glioblastoma in vivo	Chakrabarti et al.	2015	10.1007/s10495-015-1198-x	TRUE
4	Anticancer activity of flavonoids isolated from Achyrocline satureioides in gliomas cell lines	De Souza et al.	2018	10.1016/j.tiv.2018.04.013	TRUE
5	Artesunate Enhances the Antiproliferative Effect of Temozolomide on U87MG and A172 Glioblastoma Cell Lines	Karpel-Massler et al.	2014	10.2174/18715206113136660340	TRUE
6	ATM inhibitor KU-55933 increases the TMZ responsiveness of only inherently TMZ sensitive GBM cells	Nadkarni et al.	2012	10.1007/s11060-012-0979-0	TRUE
7	Berberine induces senescence of human glioblastoma cells by downregulating the EGFR-MEK-ERK signaling pathway	Liu et al.	2014	10.1158/1535-7163.MCT-14-0634	TRUE
8	beta-elemene enhances both radiosensitivity and chemosensitivity of glioblastoma cells through the inhibition of the ATM signaling pathway	Liu et al.	2015	10.3892/or.2015.4050	TRUE
9	Blocking LDHA glycolytic pathway sensitizes glioblastoma cells to radiation and temozolomide	Koukourakis et al.	2017	10.1016/j.bbrc.2017.07.138	TRUE
10	Bortezomib inhibits growth and sensitizes glioma to temozolomide (TMZ) via down-regulating the FOXM1-Survivin axis	Tang et al.	2019	10.1186/s40880-019-0424-2	TRUE

11	Bortezomib overcomes MGMT-related resistance of glioblastoma cell lines to temozolomide in a schedule-dependent manner	Vlachostergios et al.	2013	10.1007/s10637-013-9968-1	TRUE
12	Bufothionine Promotes Apoptosis via Triggering ER Stress and Synergizes with Temozolomide in Glioblastoma Multiforme Cells	Sun et al.	2019	10.1002/ar.24194	TRUE
13	Calpain suppresses cell growth and invasion of glioblastoma multiforme by producing the cleavage of filamin A	Cai et al.	2020	10.1007/s10147-020-01636-7	TRUE
14	Chemotherapeutic effect of tamoxifen on temozolomide-resistant gliomas	He et al.	2015	10.1097/CAD.0000000000000197	TRUE
15	Chloroquine enhances temozolomide cytotoxicity in malignant gliomas by blocking autophagy	Golden et al.	2014	10.3171/2014.9.FOCUS14504	TRUE
16	Chronic exposure of human glioblastoma tumors to low concentrations of a pesticide mixture induced multidrug resistance against chemotherapy agents	Doganlar et al.	2020	10.1016/j.ecoenv.2020.110940	TRUE
17	Combination of biochanin a and temozolomide impairs tumor growth by modulating cell metabolism in glioblastoma multiforme	Desai et al.	2019	10.21873/anticanres.13079	TRUE
18	Combination of caspase transfer using the human telomerase reverse transcriptase promoter and conventional therapies for malignant glioma cells	Takeuchi et al.	2004	10.3892/ijo.25.1.57	TRUE
19	Combination of the mTOR inhibitor RAD001 with temozolomide and radiation effectively inhibits the growth of glioblastoma cells in culture	Burckel et al.	2014	10.3892/or.2014.3590	TRUE
20	Combined effects of mesenchymal stem cells carrying cytosine deaminase gene with 5-fluorocytosine and temozolomide in orthotopic glioma model	Chang et al.	2020		TRUE

21	CtIP contributes to non-homologous end joining formation through interacting with ligase IV and promotion of TMZ resistance in glioma cells	Yang et al.	2019	10.26355/eurrev_201903_17252	TRUE
22	Cytotoxicity of temozolomide on human glioblastoma cells is enhanced by the concomitant exposure to an extremely low-frequency electromagnetic field (100 Hz 100 G)	Akbarnejad et al.	2017	10.1016/j.biopha.2017.05.050	TRUE
23	Development of transferrin-modified poly(lactic-co-glycolic acid) nanoparticles for glioma therapy	Mao et al.	2019	10.1097/CAD.0000000000000754	TRUE
24	Do Anti-Oxidants Vitamin D(3) Melatonin and Alpha-Lipoic Acid Have Synergistic Effects with Temozolomide on Cultured Glioblastoma Cells?	McConnell et al.	2018	10.3390/medicines5020058	TRUE
25	Down-Regulation of AQP4 Expression via p38 MAPK Signaling in Temozolomide-Induced Glioma Cells Growth Inhibition and Invasion Impairment	Chen et al.	2017	10.1002/jcb.26176	TRUE
26	Effect of the STAT3 inhibitor STX-0119 on the proliferation of a temozolomide-resistant glioblastoma cell line	Ashizawa et al.	2014	10.3892/ijo.2014.2439	TRUE
27	Effects of solvent used for fabrication on drug loading and release kinetics of electrosprayed temozolomide-loaded PLGA microparticles for the treatment of glioblastoma	Rodriguez de Anda et al.	2019	10.1002/jbm.b.34324	TRUE
28	Effects of temozolomide (TMZ) on the expression and interaction of heat shock proteins (HSPs) and DNA repair proteins in human malignant glioma cells	Castro et al.	2014	10.1007/s12192-014-0537-0	TRUE
29	EGCG inhibits properties of glioma stem-like cells and synergizes with temozolomide through downregulation of P-glycoprotein inhibition	Zhang et al.	2014	10.1007/s11060-014-1604-1	TRUE

30	EMAP-II sensitize U87MG and glioma stem-like cells to temozolomide via induction of autophagy-mediated cell death and G2/M arrest	Yu et al.	2017	10.1080/15384101.2017.1315492	TRUE
31	Enhancing glioblastoma cell sensitivity to chemotherapeutics: A strategy involving survivin gene silencing mediated by gemini surfactant-based complexes	Cruz et al.	2016	10.1016/j.ejpb.2016.04.014	TRUE
32	Exogenous IGFBP-2 promotes proliferation invasion and chemoresistance to temozolomide in glioma cells via the integrin beta 1-ERK pathway	Han et al.	2014	10.1038/bjc.2014.435	TRUE
33	Fever-Range Hyperthermia vs. Hypothermia Effect on Cancer Cell Viability Proliferation and HSP90 Expression	Kalamida et al.	2015	10.1371/journal.pone.0116021	TRUE
34	FTY720 inhibits the Nrf2/ARE pathway in human glioblastoma cell lines and sensitizes glioblastoma cells to temozolomide	Zhang et al.	2017	10.1016/j.pharep.2017.07.003	TRUE
35	G3BP1 knockdown sensitizes U87 glioblastoma cell line to Bortezomib by inhibiting stress granules assembly and potentializing apoptosis	Bittencourt et al.	2019	10.1007/s11060-019-03252-6	TRUE
36	GADD45A plays a protective role against temozolomide treatment in glioblastoma cells	Wang et al.	2017	10.1038/s41598-017-06851-3	TRUE
37	Gene expression profiling predicts response to temozolomide in malignant gliomas	Yoshino et al.	2010	10.3892/ijo_00000621	TRUE
38	Genomic profiling of long non-coding RNA and mRNA expression associated with acquired temozolomide resistance in glioblastoma cells	Zheng et al.	2017	10.3892/ijo.2017.4033	TRUE
39	Glucosylceramide synthase silencing combined with the receptor tyrosine kinase inhibitor axitinib as a new multimodal strategy for glioblastoma	Morais et al.	2019	10.1093/hmg/ddz152	TRUE

40	Growth Inhibitory Effects of Dipotassium Glycyrrhizinate in Glioblastoma Cell Lines by Targeting MicroRNAs Through the NF-kappa B Signaling Pathway	Bonafe et al.	2019	10.3892/ijmm.2015.2312	TRUE
41	Heterogeneous glioblastoma cell cross-talk promotes phenotype alterations and enhanced drug resistance	Motaln et al.	2015	10.18632/oncotarget.5701	TRUE
42	High-throughput screening uncovers miRNAs enhancing glioblastoma cell susceptibility to tyrosine kinase inhibitors	Cunha et al.	2017	10.1093/hmg/ddx323	TRUE
43	Honokiol enhances temozolomide-induced apoptotic insults to malignant glioma cells via an intrinsic mitochondrion-dependent pathway	Chio et al.	2018	10.1016/j.phymed.2018.06.012	TRUE
44	IDH1 R132H mutation regulates glioma chemosensitivity through Nrf2 pathway	Li et al.	2017	10.18632/oncotarget.15868	TRUE
45	Improved effects of honokiol on temozolomide-induced autophagy and apoptosis of drug-sensitive and -tolerant glioma cells	Chio et al.	2018	10.1186/s12885-018-4267-z	TRUE
46	In vitro and in vivo effect of human lactoferrin on glioblastoma growth	Arcella et al.	2015	10.3171/2014.12.JNS14512	TRUE
47	In vitro novel combinations of psychotropics and anti-cancer modalities in U87 human glioblastoma cells	Tzadok et al.	2010	10.3892/ijo-00000756	TRUE
48	In vitro radiosensitizing effects of temozolomide on U87MG cell lines of human glioblastoma multiforme	Borhani et al.	2017		TRUE
49	Induction of microRNA-146a is involved in curcumin-mediated enhancement of temozolomide cytotoxicity against human glioblastoma	Wu et al.	2015	10.3892/mmr.2015.4087	TRUE
50	Inhibition of STAT3 reverses alkylator resistance through modulation of the AKT and beta-catenin signaling pathways	Wang et al.	2011	10.3892/or.2011.1396	TRUE

51	Inhibition of telomerase activity in malignant glioma cells correlates with their sensitivity to temozolomide	Kanzawa et al.	2003	10.1038/sj.bjc.6601193	TRUE
52	Lithium enhances the antitumour effect of temozolomide against TP53 wild-type glioblastoma cells via NFAT1/FasL signalling	Han et al.	2017	10.1038/bjc.2017.89	TRUE
53	Magnolol and honokiol exert a synergistic anti-tumor effect through autophagy and apoptosis in human glioblastomas	Cheng et al.	2016	10.18632/oncotarget.8674	TRUE
54	Major Contribution of Caspase-9 to Honokiol-Induced Apoptotic Insults to Human Drug-Resistant Glioblastoma Cells	Wu et al.	2020	10.3390/molecules25061450	TRUE
55	Mechanisms and antitumor activity of a binary EGFR/DNA-targeting strategy overcomes resistance of glioblastoma stem cells to temozolomide	Sharifi et al.	2019	10.1158/1078-0432.CCR-19-0955	TRUE
56	MicroRNA-182 targets protein phosphatase 1 regulatory inhibitor subunit 1C in glioblastoma	Liu et al.	2017	10.18632/oncotarget.21309	TRUE
57	MicroRNA-21 silencing enhances the cytotoxic effect of the antiangiogenic drug sunitinib in glioblastoma	Costa et al.	2013	10.1093/hmg/dds496	TRUE
58	MicroRNA-29b promotes cell sensitivity to Temozolomide by targeting STAT3 in glioma	Xu et al.	2020	10.26355/eurrev_202002_20370	TRUE
59	MIM1 the Mcl-1 - specific BH3 mimetic induces apoptosis in human U87MG glioblastoma cells	Respondek et al.	2018	10.1016/j.tiv.2018.08.007	TRUE
60	MiR-144 overexpression as a promising therapeutic strategy to overcome glioblastoma cell invasiveness and resistance to chemotherapy	Cardoso et al.	2019	10.1093/hmg/ddz099	TRUE
61	miR-203 sensitizes glioma cells to temozolomide and inhibits glioma cell invasion by targeting E2F3	Tang et al.	2015	10.3892/mmr.2014.3101	TRUE
62	MiR-519a enhances chemosensitivity and promotes autophagy in glioblastoma by targeting STAT3/Bcl2 signaling	Li et al.	2018	10.1186/s13045-018-0618-0	TRUE

	pathway				
63	Mitochondria Transcription Factor A: A Putative Target for the Effect of Melatonin on U87MG Malignant Glioma Cell Line	Franco et al.	2018	10.3390/molecules23051129	TRUE
64	N-(2-hydroxyphenyl)acetamide (NA-2) and Temozolomide synergistically induce apoptosis in human glioblastoma cell line U87	Hanif et al.	2014	10.1186/s12935-014-0133-5	TRUE
65	Polyphyllin VII Promotes Apoptosis and Autophagic Cell Death via ROS-Inhibited AKT Activity and Sensitizes Glioma Cells to Temozolomide	Pang et al.	2019	10.1155/2019/1805635	TRUE
66	Quercetin sensitizes human glioblastoma cells to temozolomide in vitro via inhibition of Hsp27	Sang et al.	2014	10.1038/aps.2014.22	TRUE
67	Radiobiological evaluation and correlation with the local effect model (LEM) of carbon ion radiation therapy and temozolomide in glioblastoma cell lines	Combs et al.	2008	10.1080/09553000802641151	TRUE
68	Receptor-mediated PLGA nanoparticles for glioblastoma multiforme treatment	Ramalho et al.	2018	10.1016/j.ijpharm.2018.04.062	TRUE
69	Regulation of Integrated Stress Response Sensitizes U87MG Glioblastoma Cells to Temozolomide Through the Mitochondrial Apoptosis Pathway	He et al.	2018	10.1002/ar.23839	TRUE
70	Riluzole enhances the antitumor effects of temozolomide via suppression of MGMT expression in glioblastoma	Yamada et al.	2020	10.3171/2019.12.JNS192682	TRUE
71	Salvianolic acid B renders glioma cells more sensitive to radiation via Fis-1-mediated mitochondrial dysfunction	Chen et al.	2018	10.1016/j.biopha.2018.08.113	TRUE
72	Sequence-dependent synergistic inhibition of human glioma cell lines by combined temozolomide and miR-21 inhibitor gene therapy	Qian et al.	2012	10.1021/mp3002039	TRUE

73	Sequential treatment of phenethyl isothiocyanate increases sensitivity of temozolomide resistant glioblastoma cells by decreasing expression of mgmt via nf-kappab pathway	Guo et al.	2019		TRUE
74	Silencing SATB1 overcomes temozolomide resistance by downregulating MGMT expression and upregulating SLC22A18 expression in human glioblastoma cells	Yang et al.	2018	10.1038/s41417-018-0040-3	TRUE
75	Sirtuin 1 knockdown inhibits glioma cell proliferation and potentiates temozolomide toxicity via facilitation of reactive oxygen species generation	Chen et al.	2019	10.3892/ol.2019.10235	TRUE
76	STAT3 Inhibition Overcomes Temozolomide Resistance in Glioblastoma by Downregulating MGMT Expression	Kohsaka et al.	2012	10.1158/1535-7163.MCT-11-0801	TRUE
77	Study on therapeutic action and mechanism of TMZ Combined with RITA against glioblastoma	Wu et al.	2018	10.1159/000495923	TRUE
78	Suppression of the Eag1 potassium channel sensitizes glioblastoma cells to injury caused by temozolomide	Sales et al.	2016	10.3892/ol.2016.4992	TRUE
79	Synergistic inhibition of human glioma cell line by temozolomide and PAMAM-mediated miR-21i	Qian et al.	2012	10.1002/app.37823	TRUE
80	Synergistic suppression of noscapine and conventional chemotherapeutics on human glioblastoma cell growth	QI et al.	2013	10.1038/aps.2013.40	TRUE
81	Targeted Brain Tumor Therapy by Inhibiting the MDM2 Oncogene: In Vitro and In Vivo Antitumor Activity and Mechanism of Action	Punganuru et al.	2020	10.3390/cells9071592	TRUE
82	TAZ promotes temozolomide resistance by upregulating MCL-1 in human glioma cells	Tian et al.	2015	10.1016/j.bbrc.2015.05.115	TRUE
83	Temozolomide Cocrystals Exhibit Drug Sensitivity in Glioblastoma Cells	Kusuma et al.	2014	10.1007/s40010-014-0142-8	TRUE

84	Temozolomide induces autophagy via ATM-AMPK-ULK1 pathways in glioma	Zou et al.	2014	10.3892/mmr.2014.2151	TRUE
85	The DNA repair protein ALKBH2 mediates temozolomide resistance in human glioblastoma cells	Johannessen et al.	2012	10.1093/neuonc/nos301	TRUE
86	The Effect of Ascorbic Acid over the Etoposide- and Temozolomide-Mediated Cytotoxicity in Glioblastoma Cell Culture: A Molecular Study	Gokturk et al.	2018	10.5137/1019-5149.JTN.19111-16.1	TRUE
87	The effect of polysaccharides from Cibotium barometz on enhancing temozolomide-induced glutathione exhausted in human glioblastoma U87 cells as revealed by H-1 NMR metabolomics analysis	Shi et al.	2020	10.1016/j.ijbiomac.2020.03.243	TRUE
88	The effect of silibinin in enhancing toxicity of temozolomide and etoposide in p53 and PTEN-mutated resistant glioma cell lines	Elhag et al.	2015		TRUE
89	The Effect of Temozolomide/Poly(lactide-co-glycolide) (PLGA)/Nano-Hydroxyapatite Microspheres on Glioma U87 Cells Behavior	Zhang et al.	2012	10.3390/ijms13011109	TRUE
90	The HIV-derived protein Vpr52-96 has anti-glioma activity in vitro and in vivo	Kübler et al.	2016	10.1158/1538-7445.AM2015-4458	TRUE
91	The mTOR inhibitor RAD001 potentiates autophagic cell death induced by temozolomide in a glioblastoma cell line	Josset et al.	2013		TRUE
92	The Pan-Bcl-2 Inhibitor (-)-Gossypol Triggers Autophagic Cell Death in Malignant Glioma	Voss et al.	2010	10.1158/1541-7786.MCR-09-0562	TRUE
93	The Synergistic Effect of Combination Progesterone and Temozolomide on Human Glioblastoma Cells	Atif et al.	2015	10.1371/journal.pone.0131441	TRUE
94	The synergistic effect of combination temozolomide and chloroquine treatment is dependent on autophagy formation and p53 status in glioma cells	Lee et al.	2015	10.1016/j.canlet.2015.02.012	TRUE

95	Tim-3 expression in glioma cells is associated with drug resistance	Zhang et al.	2019	10.4103/jcrt.JCRT_630_18	TRUE
96	Tramadol attenuates the sensitivity of glioblastoma to temozolomide through the suppression of Cx43-mediated gap junction intercellular communication	Wang et al.	2018	10.3892/ijo.2017.4188	TRUE
97	Transcriptional targeting of adenovirally delivered tumor necrosis factor alpha by temozolomide in experimental glioblastoma	Yamini et al.	2004	10.1158/0008-5472.CAN-04-2117	TRUE
98	Verapamil potentiates anti-glioblastoma efficacy of temozolomide by modulating apoptotic signaling	Hanif et al.	2018	10.1016/j.tiv.2018.07.001	TRUE
99	Verbascoside inhibits glioblastoma cell proliferation migration and invasion while promoting apoptosis through upregulation of protein tyrosine phosphatase SHP-1 and inhibition of STAT3 phosphorylation	Jia et al.	2018	10.1159/000491067	TRUE
100	Zinc enhances TMZ cytotoxicity in glioblastoma multiforme model system	Toren et al.	2016	10.1093/neuonc/not176	TRUE
101	β-Elemene inhibits proliferation through crosstalk between glia maturation factor β and extracellular signal-regulated kinase 1/2 and impairs drug resistance to temozolomide in glioblastoma cells	Zhu et al.	2014	10.3892/mmr.2014.2273	TRUE
102	Anti-epidermal growth factor receptor siRNA carried by chitosan-transacylated lipid nanocapsules increases sensitivity of glioblastoma cells to temozolomide	Messaoudi et al.	2014	10.2147/IJN.S59134	FALSE
103	Autophagic flux response and glioblastoma sensitivity to radiation	Mitrakas et al.	2018	10.20892/j.issn.2095-3941.2017.0173	FALSE
104	Autophagy mediates glucose starvation-induced glioblastoma cell quiescence and chemoresistance through coordinating cell metabolism cell	Wang et al.	2018	10.1038/s41419-017-0242-x	FALSE

	cycle and survival				
105	BET inhibitor I-BET151 sensitizes GBM cells to temozolomide via PUMA induction	Yao et al.	2019	10.1038/s41417-018-0068-4	FALSE
106	Combined effect of 2-5A-linked antisense against telomerase RNA and conventional therapies on human malignant glioma cells in vitro and in vivo	Iwado et al.	2007	10.3892/ijo.31.5.1087	FALSE
107	Decreasing GSH and increasing ROS in chemosensitivity gliomas with IDH1 mutation	Shi et al.	2014	10.1007/s13277-014-2644-z	FALSE
108	Downregulation of Id2 increases chemosensitivity of glioma	Zhao et al.	2015	10.1007/s13277-015-3055-5	FALSE
109	Downregulation of miR-155 inhibits proliferation and enhances chemosensitivity to Temozolomide in glioma cells	Meng et al.	2017		FALSE
110	Downregulation of miR-196b Promotes Glioma Cell Sensitivity to Temozolomide Chemotherapy and Radiotherapy	Ma et al.	2018		FALSE
111	Effect of temozolomide on the viability of musculoskeletal sarcoma cells	Kusabe et al.	2015	10.3892/ol.2015.3506	FALSE
112	Effects of galbanic acid on proliferation migration and apoptosis of glioblastoma cells through PI3K/Akt/mTOR signaling pathway	Shahcheraghi et al.	2020	10.2174/1874467213666200512075507	FALSE
113	Encapsulation of Temozolomide in a Calixarene Nanocapsule Improves Its Stability and Enhances Its Therapeutic Efficacy against Glioblastoma	Renziehausen et al.	2019	10.1158/1535-7163.MCT-18-1250	FALSE
114	FTO inhibition enhances the anti-tumor effect of temozolomide by targeting MYC-miR-155/23a cluster-MXI1 feedback circuit in glioma	Xiao et al.	2020	10.1158/0008-5472.CAN-20-0132	FALSE
115	Green tea epigallocatechin gallate enhances therapeutic efficacy of temozolomide in orthotopic mouse	Chen et al.	2011	10.1016/j.canlet.2010.11.008	FALSE

	glioblastoma models				
116	Growth-inhibitory and chemosensitizing effects of microRNA-31 in human glioblastoma multiforme cells	Zhou et al.	2015	10.3892/ijmm.2015.2312	FALSE
117	Growth-inhibitory and chemosensitizing effects of the glutathione-S- transferase-pi-activated nitric oxide donor PABA/NO in malignant gliomas	Kogias et al.	2012	10.1002/ijc.26106	FALSE
118	Identification of Key Candidate Proteins and Pathways Associated with Temozolomide Resistance in Glioblastoma Based on Subcellular Proteomics and Bioinformatical Analysis	Yi et al.	2018	10.1155/2018/5238760	FALSE
119	Inhibition of EZH2 reverses chemotherapeutic drug TMZ chemosensitivity in glioblastoma	Fan et al.	2014		FALSE
120	Inhibition of JNK Potentiates Temozolomide-induced Cytotoxicity in U87MG Glioblastoma Cells via Suppression of Akt Phosphorylation	Vo et al.	2014		FALSE
121	Long noncoding RNA RP11-838N2.4 enhances the cytotoxic effects of temozolomide by inhibiting the functions of miR-10a in glioblastoma cell lines	Liu et al.	2016	10.18632/oncotarget.9699	FALSE
122	LY294002 enhances cytotoxicity of temozolomide in glioma by down-regulation of the PI3K/Akt pathway	Chen et al.	2012	10.3892/mmr.2011.674	FALSE
123	Mechanisms operative in the antitumor activity of temozolomide in glioblastoma multiforme	Fischer et al.	2007	10.1097/PPO.0b013e318157053f	FALSE
124	MiR-21 protected human glioblastoma U87MG cells from chemotherapeutic drug temozolomide induced apoptosis by decreasing Bax/Bcl-2 ratio and caspase-3 activity	Shi et al.	2010	10.1016/j.brainres.2010.07.009	FALSE
125	Mutant TP53 enhances the resistance of glioblastoma cells to temozolomide by up-regulating O-	Wang et al.	2012	10.1007/s10072-012-1257-9	FALSE

	6-methylguanine DNA-methyltransferase				
126	Next Generation Sequencing-Based Transcriptome Predicts Bevacizumab Efficacy in Combination with Temozolomide in Glioblastoma	Adilijiang et al.	2019	10.3390/molecules24173046	FALSE
127	Olanzapine inhibits proliferation migration and anchorage-independent growth in human glioblastoma cell lines and enhances temozolomide's antiproliferative effect	Karpel-Massler et al.	2014	10.1007/s11060-014-1688-7	FALSE
128	Ovatodiolide inhibits the oncogenicity and cancer stem cell-like phenotype of glioblastoma cells as well as potentiate the anticancer effect of temozolomide	Su et al.	2019	10.1016/j.phymed.2019.152840	FALSE
129	Overexpression of iASPP-SV in glioma is associated with poor prognosis by promoting cell viability and antagonizing apoptosis	Liu et al.	2015	10.1007/s13277-015-4503-y	FALSE
130	Polymer - Temozolomide Conjugates as Therapeutics for Treating Glioblastoma	Ward et al.	2018	10.1021/acs.molpharmaceut.8b00766	FALSE
131	Potentiation of antiglioma effect with combined temozolomide and interferon-beta	Park et al.	2006		FALSE
132	Silence of bFGF enhances chemosensitivity of glioma cells to temozolomide through the MAPK signal pathway	Wang et al.	2016	10.1093/abbs/gmw035	FALSE
133	Synergistic combination of chemo-phototherapy based on temozolomide/ICG-loaded iron oxide nanoparticles for brain cancer treatment	Kwon et al.	2019	10.3892/or.2019.7289	FALSE
134	Temozolomide Gemcitabine and Decitabine Hybrid Nanoconjugates: From Design to Proof-of-Concept (PoC) of Synergies toward the Understanding of Drug Impact on Human Glioblastoma Cells	Sahli et al.	2020	10.1021/acs.jmedchem.0c00694	FALSE

135	The synergic antitumor effects of paclitaxel and temozolomide co-loaded in mPEG-PLGA nanoparticles on glioblastoma cells	Xu et al.	2016	10.18632/oncotarget.7896	FALSE
136	Vincristine and temozolomide combined chemotherapy for the treatment of glioma: a comparison of solid lipid nanoparticles and nanostructured lipid carriers for dual drugs delivery	Wu et al.	2015	10.3109/10717544.2015.1058434	FALSE
137	YKL-40 downregulation is a key factor to overcome temozolomide resistance in a glioblastoma cell line	Akiyama et al.	2014	10.3892/or.2014.3195	FALSE

Extracted data for every included article are provided in the supplementary datasets that are available on GitHub (https://github.com/TimoSander/Reporting-practices-as-a-source-of-heterogeneity-in-in-vitro-cancer-research/).

A7: Reported temozolomide concentrations in the included studies into meta-analysis

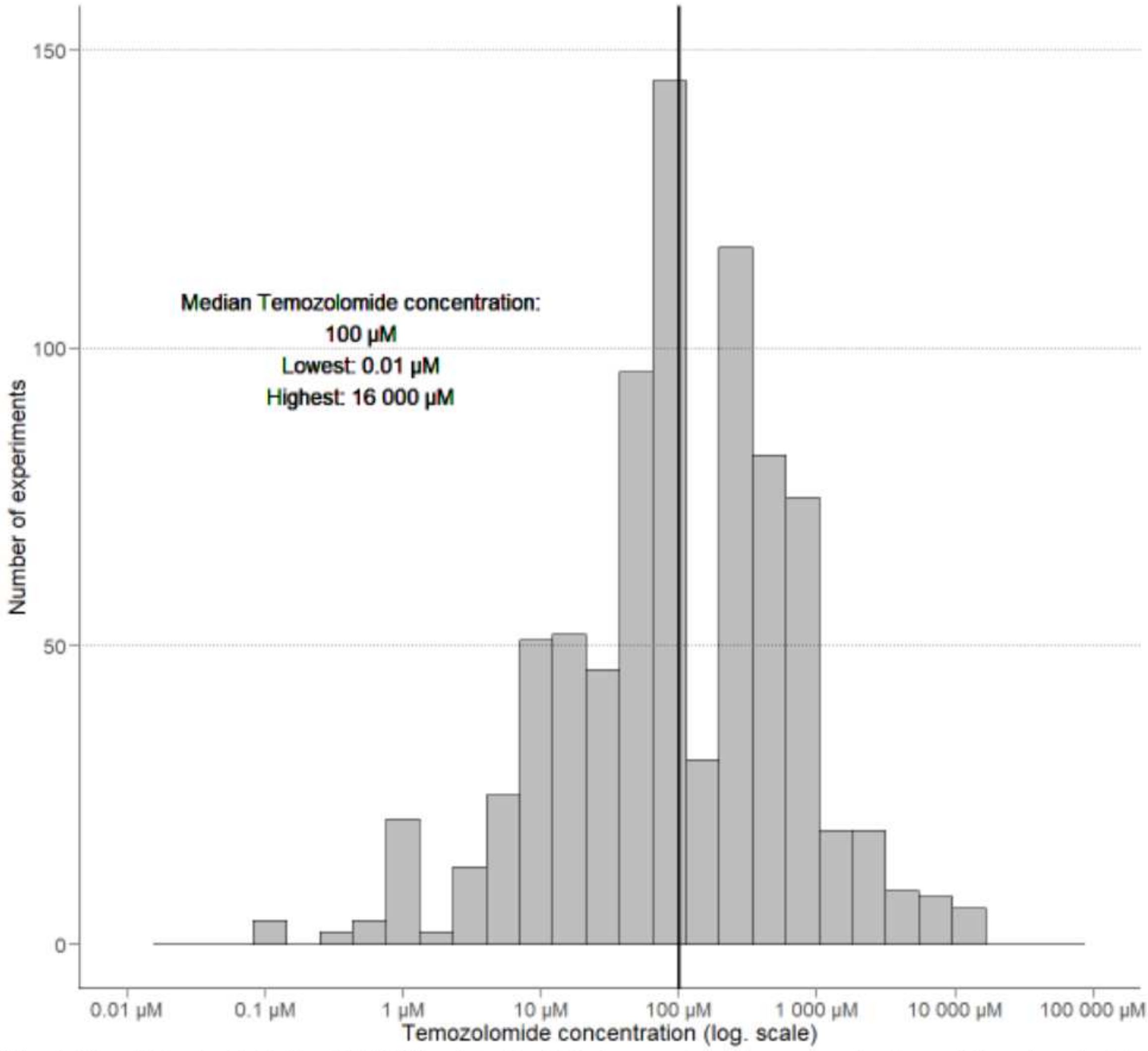

The 137 articles included cell viability data for 828 temozolomide concentrations (98 unique). The drug's concentration is presented on a logarithmic scale.

A8: Reported treatment durations in the included studies into meta-analysis

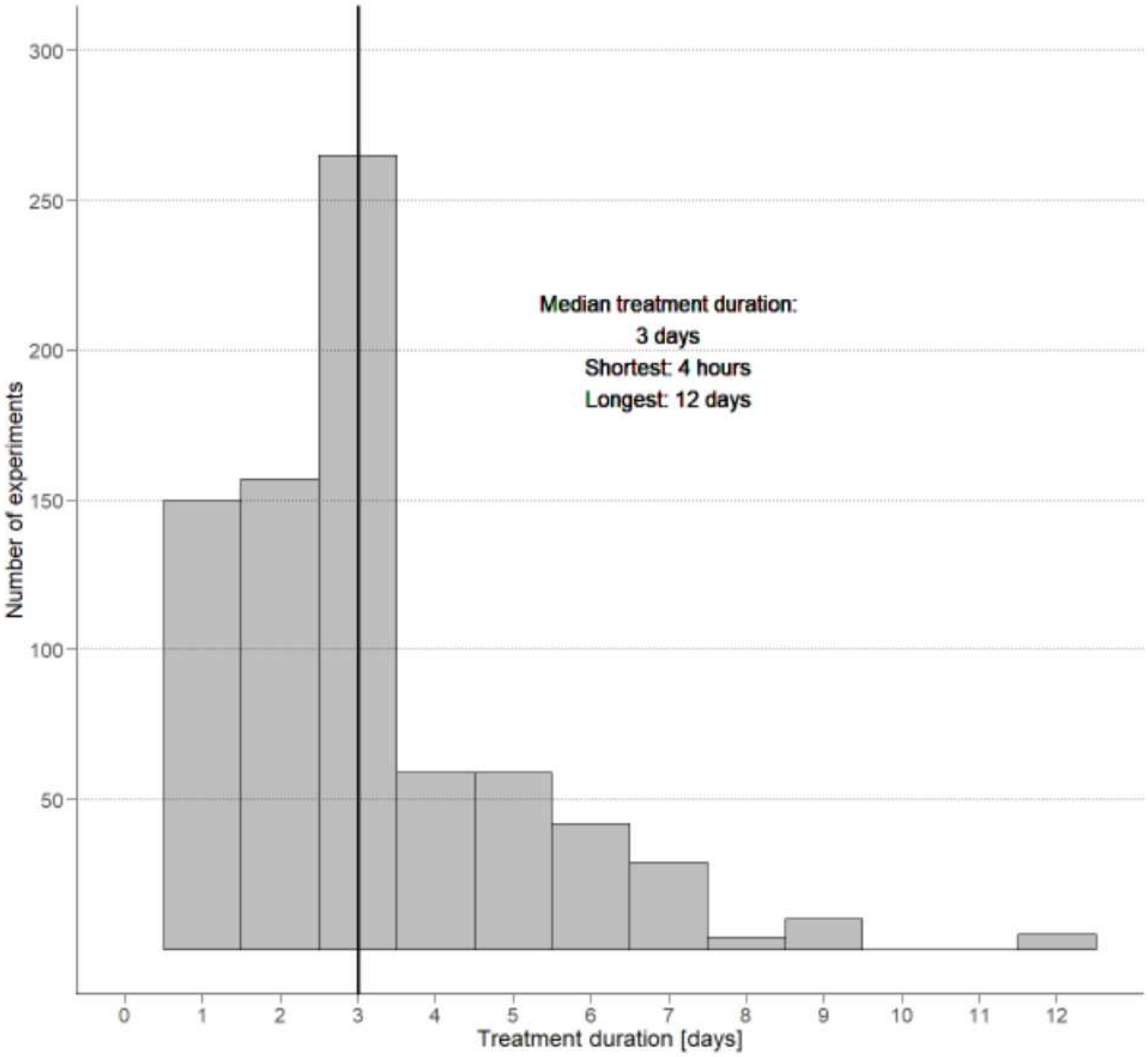

The 137 articles included cell viability data for 786 durations of temozolomide exposure (20 unique). The lower number in comparison to used temozolomide concentrations is due to non-reporting. Treatment duration was defined as the duration of exposure of Uppsala-87 Malignant Glioma cells to temozolomide.

A9: Risk of bias factors prevalence

Potential risk of bias	Articles	
Missing sample size calculation	137	100.0%
No random group allocation	137	100.0%
No blinded outcome assessment	137	100.0%
No open-access study protocol available	137	100.0%
Unclear way of calculation for cell viability average and error values	92	67.2%
Unclear number of independent experiments and replications per experiment	47	34.3%
Data were not presented for every experiment	12	8.8%
Missing data (for particular drug concentrations and/or treatment durations) within an experiment	1	0.7%

The column "articles" shows the absolute and relative prevalence of articles having a particular risk of bias factor in comparison to all 137 included articles

A10: Literature flow chart of systematic search update

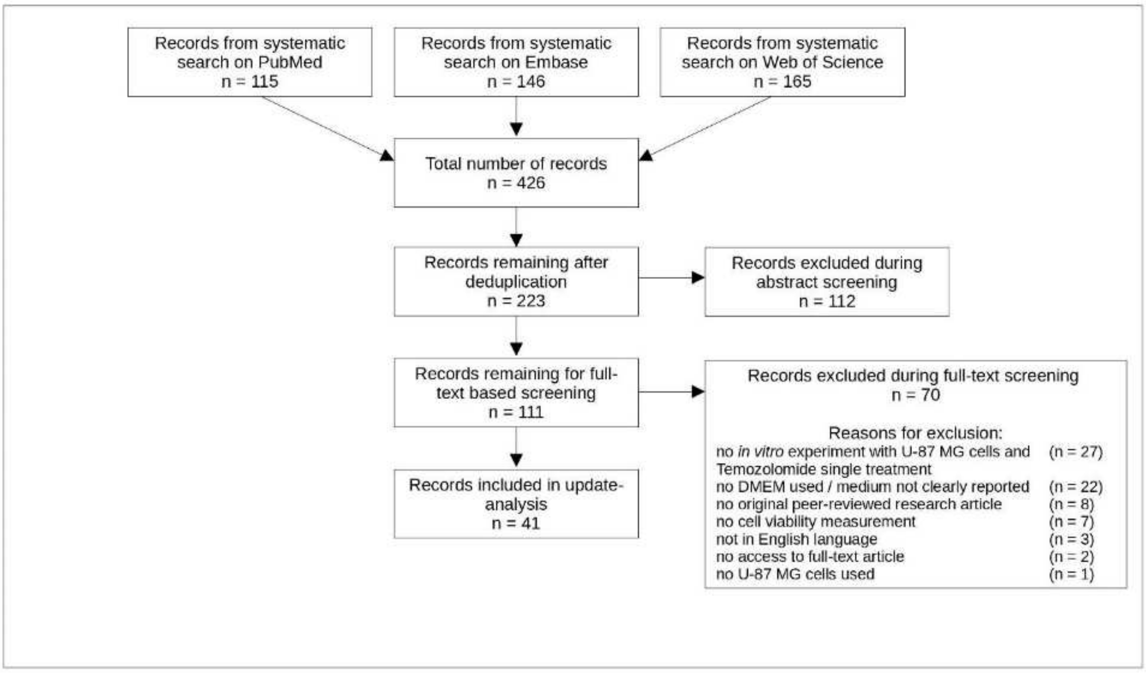

Presentation based on the PRISMA statement (2020). Updated systematic search was conducted on 10th of March 2022. One reason for exclusion per excluded article. DMEM = Dulbecco's modified Eagle's medium; U-87 MG = Uppsala-87 Malignant Glioma.

A11: Updated assessment of reporting and risks of bias

	**Study parameters' reporting quality**			
**No.**	**Parameter**	**Reporting 09/2020 – 03/2022**	**Reporting until 08/2020**	**Change**
1	Source of U-87 MG	95.1%	94.2%	0.9%
2	Concentration of TMZ	95.1%	94.2%	0.9%
3	Errortype	85.4%	87.6%	-2.2%
4	Treatment duration	97.6%	87.6%	10.0%
5	Number of experiments	78.0%	82.5%	-4.5%
6	Source of TMZ	68.3%	73.7%	-5.4%
7	Antibiotics	78.0%	73.0%	5.0%
8	Source of FBS	73.2%	69.3%	3.9%
9	Conflicts of interest	92.7%	66.4%	26.3%
10	Type of control	29.3%	36.5%	-7.2%
11	Glucose level	19.5%	21.2%	-1.7%
12	U-87 MG concentration	34.1%	16.8%	17.3%
13	Cell passaging criteria	14.6%	12.4%	2.2%
14	U-87 MG authentication	12.2%	9.5%	2.7%
15	U-87 MG age	14.6%	7.3%	7.3%
16	Mycoplasma exclusion	9.8%	5.8%	4.0%
17	Volume of added TMZ	7.3%	5.1%	2.2%
18	Volume of added control	4.9%	2.2%	2.7%
	**Prevalence of risks of bias**			
	**Risk of bias**	**Prevalence 09/2020 – 03/2022**	**Prevalence until 08/2020**	**Change**
19	Missing sample size calculation	100.0%	100.0%	0.0%
20	No random group allocation	100.0%	100.0%	0.0%
21	No blinded outcome assessment	100.0%	100.0%	0.0%
22	No open-access study protocol available	100.0%	100.0%	0.0%
23	Unclear way of calculation for cell viability average and error values	80.5%	67.2%	13.3%
24	Unclear number of independent experiments and replications per experiment	56.1%	34.3%	21.8%
25	Data were not presented for every experiment	4.9%	8.8%	-3.9%
26	Missing data (for particular drug concentrations and/or treatment durations) within an experiment	2.4%	0.7%	1.7%

Comparison of study parameter reporting quality (share of articles that reported the parameter) and prevalence of risks of bias until the time of the systematic literature search in 03/2020 (137 articles) and thereafter until 03/2022 (41 articles). Change (%) was calculated setting reporting/prevalence of risks of bias until 08/2020 as reference point (100 %). FBS = fetal bovine serum, No. = number, TMZ = Temozolomide, U-87 MG = Uppsala 87 Malignant Glioma.

A12: Confidence intervals for the updated assessment of reporting and risks of bias

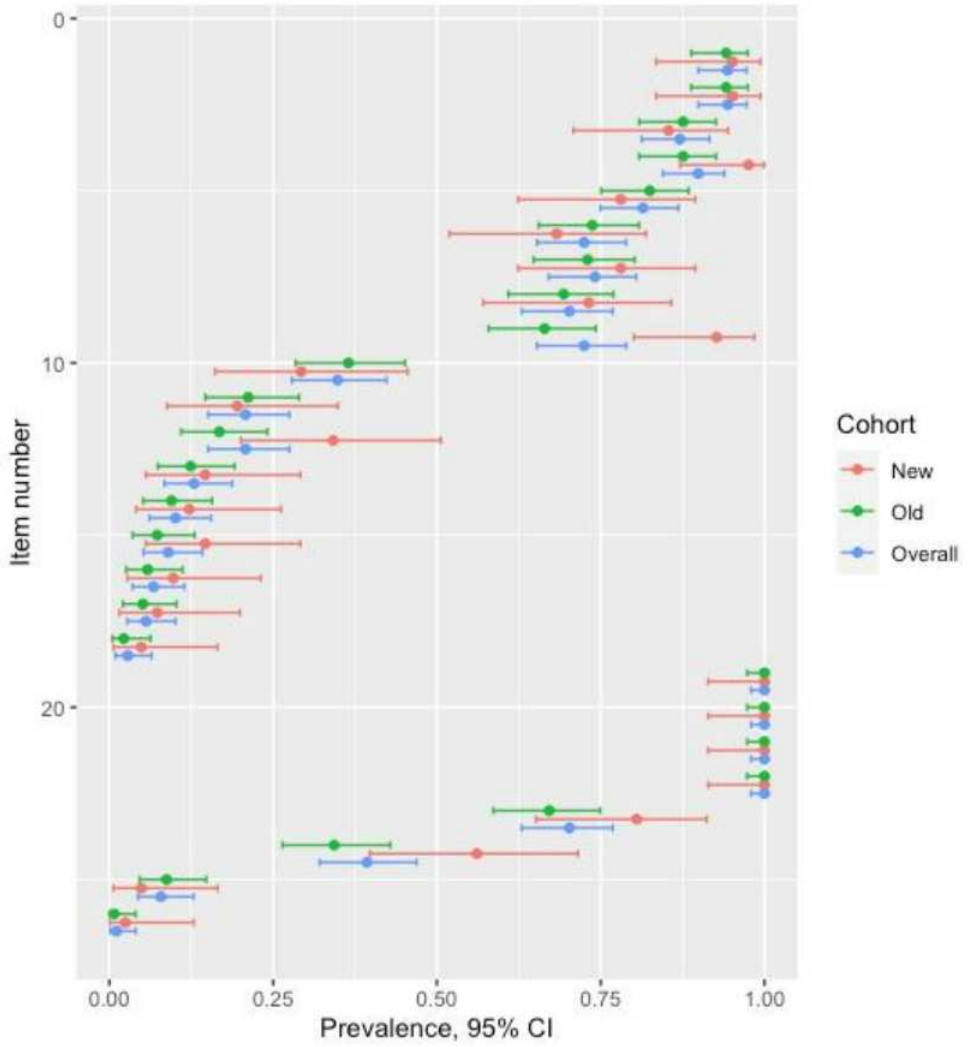

Confidence intervals (95 %) for the prevalence of reported study parameters and risks of bias. Y-axis indicates the item number according to S11. Red data indicate prevalence in the search for additional articles in the timespan of September 2020 until March 2022, green data indicate prevalence in the systematic search conducted in August 2020, and the blue data indicate the overall prevalence including old and new search results.

A13: Update on overall reporting quality of articles

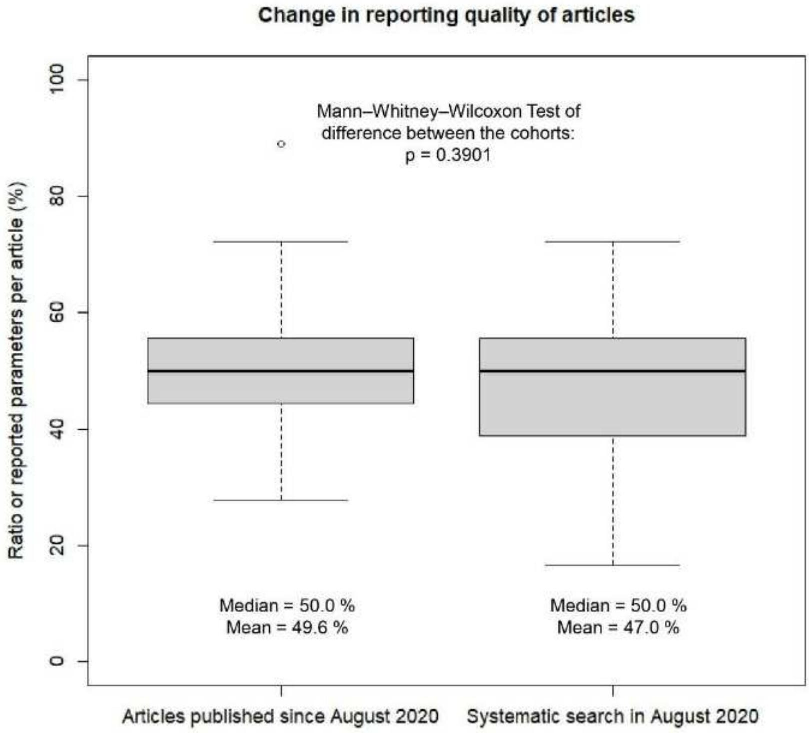

Comparison of articles reporting quality at the time points of initial systematic search in August 2020 and in March 2022. Articles reporting quality was calculated as share of reported parameters including the 18 study parameters presented in Supplement S10. Median and mean reporting qualities for both cohorts are presented underneath the boxplots. The boxplots indicate median, 25th (Q1) and 75th (Q3) percentile (interquartile range (IQR)), Q1 – 1.5 x IQR, Q3 + 1.5 x IQR, and outliers of reporting quality. Mann-Whitney-Wilcoxon Test was used for comparison of reporting across both cohorts with the null hypothesis of a no change in reporting.

A14: Update on overall risks of bias prevalence

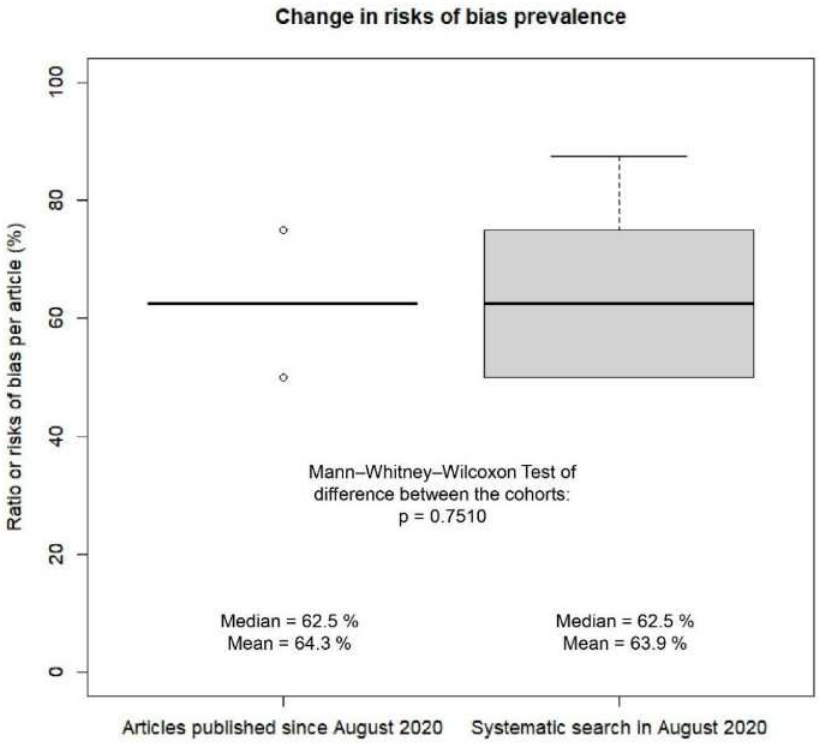

Comparison of the prevalence of risks of bias at the time points of initial systematic search in August 2020 and in March 2022. The prevalence of risks of bias was calculated as share of present risks of bias including the eight potential risk of bias parameters presented in Supplement S10. Median and mean risks of bias prevalence for both cohorts are presented underneath the boxplots. The boxplots indicate median, 25th (Q1) and 75th (Q3) percentile (interquartile range (IQR)), Q1 – 1.5 x IQR, Q3 + 1.5 x IQR, and outliers of reporting quality. Mann-Whitney-Wilcoxon Test was used for comparison of risks of bias prevalence across both cohorts with the null hypothesis of no change in prevalence.

A15: Moderators of within-articles-variance of true effects

Moderator	Type	Number of effects	Number of articles	*p* value	Marginal R^2	Within-articles-variance		
						tau²	I^2	Explained
Without moderators		644	101			3.6%	56.6%	n. a.
U-87 MG source	cat.	644	101	.075	n. s.	3.6%		
U-87 MG authentication	cat.	644	101	.476	n. s.	3.6%		
U87-MG age (Cell passages)	cont.	138	11	.238	n. s.	4.0%		
Cell concentration	cont.	113	20	.323	n. s.	4.8%		
Confluence level at cell passaging	cont.	57	11	.319	n. s.	4.2%		
Glucose level of culture medium	cat.	644	101	.016	7.0%	3.6%[a]	59.4%	0.0%
Mycoplasma exclusion	cat.	644	101	.491	n. s.	3.6%		
Supplemented antibiotics	cat.	644	101	.094	n. s.	3.6%		
FBS source	cat.	644	101	.067	n. s.	3.6%		
Type of control	cat.	644	101	.370	n. s.	3.6%		
Articles reporting quality	int.	644	101	.031	3.3%	3.6%[b]	57.8%	0.0%
TMZ conc.	cont.	644	101	< .001	38.6%	1.9%[c]	35.0%	48.3%
Treatment duration	cont.	644	101	< .001	6.0%	3.4%[d]	53.8%	4.8%

Random-effects three-level meta-regressions with the raw data the effects were calculated with as first level, the reported effects as second level and the articles the effects were reported in as third level. Marginal R^2 indicates the regression model fit [65]; tau²: estimator of the variance of true effects; tau = square root of tau²; I^2: proportion of within-articles-variance of the total observed variance including sampling error. tau² estimator: restricted-maximum likelihood. The column "explained" indicates the reduction of tau² after including the particular moderator compared to tau² without moderators (only applicable if the number of included effects and articles is identical). Types of moderators: cat. = categorical; cont. = continuous, int. = interval. For some continuous moderators, the number of effects and articles included in the regression is reduced due to non-reporting which leads to a limited comparability of tau² between parameters with different numbers of belonging articles and effects. "Not reported" was included as a category for categorical moderators. The *p* value is for the test of the moderator. R^2, I^2 and the explained heterogeneity were only calculated for moderators that prove significance in the test of the moderator (alpha = .05). TMZ conc. = Temozolomide concentration. **a**: 95%-confidence-interval (CI): tau²: [3.2%,4.1%]; **b**: 95%-CI of tau²: [3.2%,4.1%]; **c**: 95%-CI of tau²: [1.6%,2.2%]; **d**: 95%-CI of tau²: [3.0%,3.9%].

A16: Correlation between the effect and culture mediums glucose level as well as articles reporting quality

Moderator	Effects	Articles	Cell viability reduction	SD	CI LL	CI UL	*p* value
**Glucose level**							
High glucose (4500 mg/dl)	168	21	23.1%	3.6%	15.8%	30.5%	< 0.001
Low glucose (1000 mg/dl)	37	2	20.6%	5.8%	-25.2%	66.3%	.726*
No glucose	1	1	29.0%	3.5%	21.6%	36.4%	.113*
Not reported	438	77	37.1%	4.2%	28.6%	45.6%	.002*
**Articles reporting quality**							
Intercept	644	101	61.3%	12.0%	37.0%	85.7%	< .001
Change per unit increase in the number of reported parameters			-3.0%	1.3%	-5.5%	-0.4%	.026

Univariable Random-effects three-level meta-regressions with the raw data the effects were calculated with as first level, the reported effects as second level and the articles the effects were reported in as third level. Effects were estimated using robust-variance-estimation. Cell viability reduction is presented in comparison to the corresponding untreated control. A linear regression model was applied for the articles reporting quality correlation analysis. The reduced number of included articles and effects for glucose level analysis was a result of non-reporting. SD = standard deviation; CI = confidence interval (with significance level of alpha = .05); LL = lower limit; UL = upper limit. [a]: The *p* value indicates whether there was a significant difference for the parameter phenotypes effect estimate in comparison to the effect estimate in the high glucose group.
